# Autonomy and its relevance for the construction of personhood in dementia– a thematic synthesis

**DOI:** 10.1186/s12877-024-04808-6

**Published:** 2024-03-14

**Authors:** Jonathan Serbser-Koal, Jan Dreyer, Martina Roes

**Affiliations:** 1 Deutsches Zentrum für Neurodegenerative Erkrankungen (DZNE), Stockumer Str. 12, 58453 Witten, Germany; 2 Department für Pflegewissenschaft, Fakultät für Gesundheit, Universität Witten/Herdecke, Witten, Germany

**Keywords:** Dementia, Autonomy, Personhood, Person-centeredness, Relationality, Social health

## Abstract

**Background:**

This article examines the concept of autonomy in the context of person-centred dementia research and care, which is frequently being used but not clearly defined. Also, there is no clear conceptual relation between autonomy and personhood in this context.

**Methods:**

Therefore, literature on person-centred dementia research and care was examined to answer the following question: How is the concept of autonomy discussed in person-centred dementia research and care literature?

**Results:**

This analysis revealed heterogeneous perspectives on autonomy within the context of dementia. These were assigned to two different perspectives on personhood: one that links personhood to the existing cognitive abilities and the other one, that understands personhood relationally as the result of a socially constructed process. These results are discussed with regard to a nursing and care practice that could be considered as being deficit-oriented, but also with regard to the concept of social health in dementia.

**Conclusions:**

Derived from this analysis, there is a clear need for general conceptual sensitivity in this field. Also, an in-depth examination of the social constructionist approach to personhood in the context of dementia is warranted.

**Supplementary Information:**

The online version contains supplementary material available at 10.1186/s12877-024-04808-6.

## Introduction

The subject of this article is autonomy and dementia in the context of person-centred care. The connection between the two concepts in this context is not unproblematic because when dealing with the concept of autonomy in the context of dementia, the personhood of people with dementia must also be considered. The following explanations are based on the assumption that the understanding of being a person is the basis for the interpretation of the concept of autonomy. This argument initially occurs on a kind of metalevel because it involves discourses on conceptualities and their interpretation and classification. Thus, an analysis of concepts in certain contexts seems to be useful in many respects. In particular, when concepts are used particularly frequently and as a matter of course in everyday and practical usage, the suspicion of a “Konsensfiktion” [1]– which is defined as a fictitious agreement regarding its actual meaning– may arise. But also research, which is strongly practice-oriented, would do well to constantly review the terms it uses, including conceptually. Against this background, it makes sense to broaden the discourse on the terms in question and to trace the differentiation of these terms. There are also advantages in understanding practical applications because depending on how terms are understood and used, different possibilities for action arise.

In the following, we would like to pursue this idea and attempt a conceptual analysis of autonomy in the context of person-centred dementia care. First, however, we will examine the concepts that we consider to be relevant.

### Epistemological interest in person-centeredness and autonomy

The focus of our article is the concept of autonomy. Following Kotsch and Hitzler [1], the concept of self-determination, although it has been relatively vaguely defined, is frequently and naturally used in everyday life. This indicates what Kotsch and Hitzler would call a “Konsensfiktion” [1] with regard to the term. Agich [2] emphasizes that autonomy has a wide variety of connotations and that it would be a mistake to attempt to establish a uniform definition. The concept of autonomy must always be understood in relation to the particular context in which it is used. According to Agich, the fundamental issue in addressing the idea of autonomy is the question of personal identity [3]. In the standard model of autonomy, patient autonomy is often equated with an understanding in which the autonomous agent must possess certain ideal capacities: the ability to act as an independent, rational decision maker, as someone who knows his or her own desires and preferences and whose freedom is expressed in actions or decisions directed towards the fulfilment of those desires and preferences [3].

In a statement, the German Ethics Council addressed the topic of self-determination and dementia [4]. Three aspects of the philosophical concept of self-determination are defined here: (1) “To be able to act differently”, i.e., to have several options for action; (2) “To have reasons”, i.e., to make a justified choice from these options for action; and (3) “It is I”, i.e., to have an awareness of one’s own authorship. It is presupposed that the nature and scope of these three aspects are understood, that they are evaluated against the background of the individual life situation and attitude and that action is aligned with them in each case [4]. In the context of dementia, it is assumed that the person increasingly loses his or her autonomy in the course of the disease. Although restrictions are imposed because in dementia, the cognitive quality of the self is subject to change, and its emotional, social-communicative, everyday practical, sensory and aesthetic qualities continue. In addition, responsibility for one’s own actions ceases to exist only after a certain stage [4]. In the majority vote of the Ethics Council, the assumption and possibility of assisted partial autonomy, i.e., a dimensionalized or liminal concept of autonomy, follows from these considerations.

According to Aveyard [5], the concept of autonomy is interpreted and used differently in nursing practice. This is in line with the above observation by Kotsch and Hitzler [1].

Thus, the concept of autonomy in the context of dementia confronts us with a term that is frequently used but also does not seem to have a uniform definition. This leads us to the perspective that the findings of an analysis of the concept of autonomy in the context of dementia expand the debate about personhood and dementia on the basis of the aspect of cognition or cognitive ability– or, in contrast, by the aspect of relationality– and thus open up the opportunity for an elaborated understanding of the idea of personhood in the context of dementia. The perspectives on personhood are to be understood as a kind of intermediate result with which we then continue to work and differentiate the ideas on autonomy.

### Person-centredness, social health and autonomy

Since the 1990s, the concept of *person-centredness* has been considered a point of reference in dementia care [6]. However, how exactly do we understand the idea of personhood in that specific context? For Kitwood, the approach of considering personhood from the perspective of relationships was crucial. He brought his view to a fundamental term within the concept of person-centredness: “de-personalization”. This term describes the outcome of the process when the disease is seen as paramount, undermining the actual and still existing personhood of people living with dementia. To counter this process, Kitwood focused on the recognition and preservation of personhood. Subsequently, his concept has been widely expanded upon and forms the basis for many approaches to dementia care [7–10].

However, at least from a theoretical-conceptual point of view, there is the challenge that in many cases, the extent to which person-centredness can be understood as a unified concept is questionable; moreover, there is a lack of clarity about how the personhood of people living with dementia can be conceptualized and understood in this context [10–15]. To be able to answer the question of how the personhood of people living with dementia can be understood, we conducted a scoping review on person-centredness and dementia that forms the basis for the subsequent conceptual analysis of autonomy within this article. To illustrate and explain the development of the literature sample, the methodological description of the scoping review on person-centredness and dementia can be found in the additional file (see Additional file 1).

In addition to the analysis of the literature on person-centredness and dementia, it became clear that person-centredness plays a considerable role within the debate on *social health* and dementia [16–18]. Social health is understood as an overarching concept that, in addition to physical and mental health, encompasses the social aspects of health. This concept appears to be particularly important for the field of dementia, as it emphasizes– in accordance with the person-centred approach– the nonmedical side of health, focusing on *remaining* capacities rather than emphasizing deficits. In this regard, dementia can be seen as an overcomplex phenomenon because as a chronic condition, it requires equivalent confrontation in all three fields of health. Although within the last century, the focus was on the description of pathology [17], it may now be possible to shift the focus towards the social aspects of dementia. A study of the operationalization of social health relating to dementia was led by a task force of the pan-European network INTERDEM [16]. They assumed the three dimensions of social health– *capacity to fulfil one’s potential and obligations*, *managing life with some degree of independence*, and *participation in social activities*– formulated by Huber et al. [19] and differentiated them according to the requirements and demands of the implications of dementia.

In this view, the first dimension, “capacity to fulfil one’s potential and obligations”, could be achieved if people living with dementia have the capacity to exercise choice and *autonomy*, to maintain their own identity (personhood), to participate and contribute to communities, to give and receive support (reciprocity), to collaborate with professional and informal carers, to participate in shared decision-making and to participate and contribute to communities. The second dimension, “managing life with some degree of independence”, may become possible if people living with dementia can preserve autonomy and solve problems in daily life as well as adapt to and cope with the practical and emotional consequences of dementia. For the last dimension, “participation in social activities”, people living with dementia must be occupied or involved in meaningful activities and social interactions and have social ties and relationships that are meaningful to them. It is apparent that certain concepts that are addressed implicitly in the debate on social health and dementia, namely, identity, individuality, dignity, recognition, autonomy and independence, are also fundamental for person-centred dementia care. Each of these is worth further elaboration in the context of dementia.

Our epistemological interest led to our decision to conduct a secondary analysis of the literature in the field of person-centred dementia care, which was previously identified through the aforementioned scoping review (see also Additional file 1). Following the methodological concept of secondary analysis [20], we analysed the literature drawn from the scoping review in terms of the following question: How is the concept of autonomy discussed in the person-centred dementia research and care literature?

## Methods

**Fig. 1:**
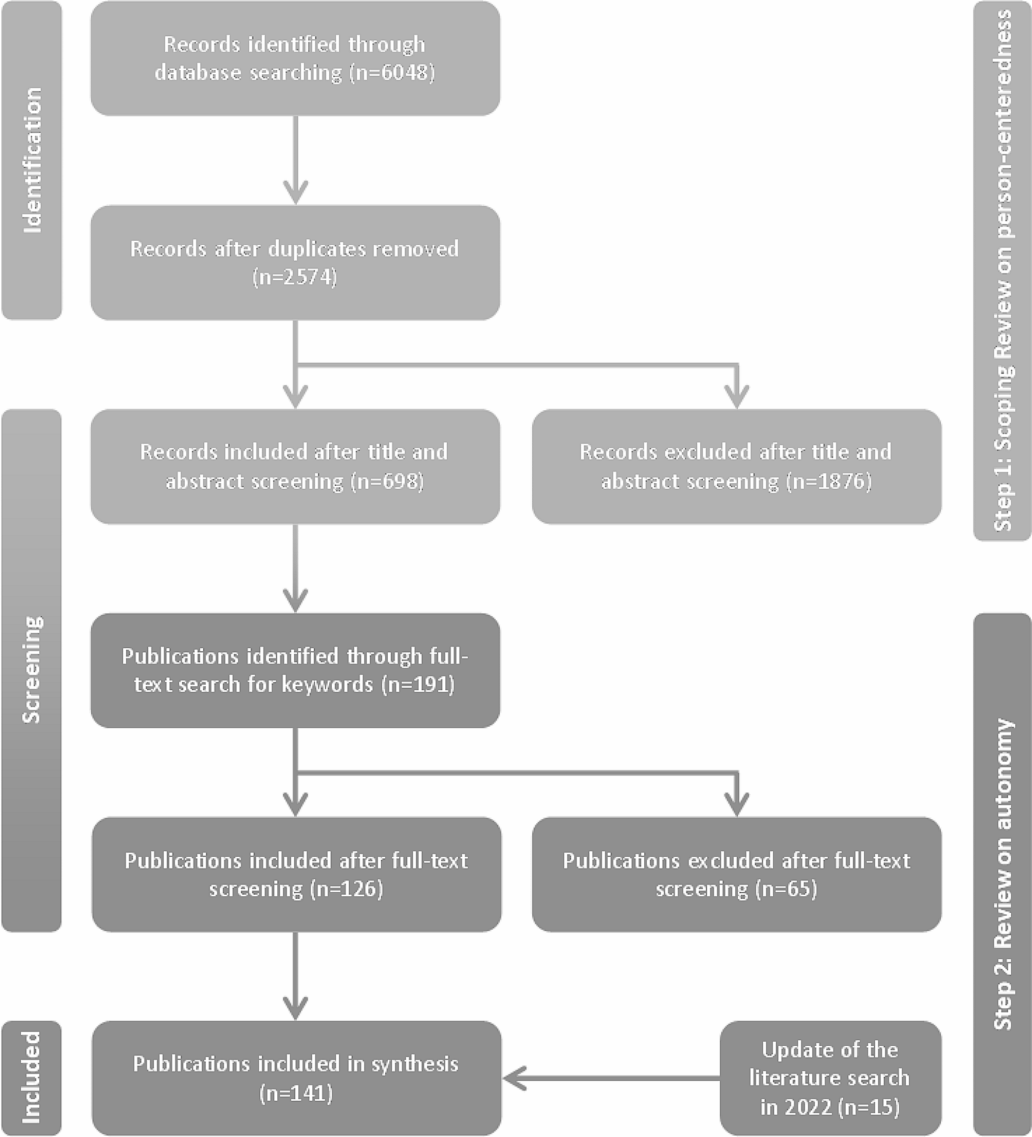
Flow diagram

To conduct the secondary analysis, we drew upon the literature that we had previously identified through a scoping review within the context of person-centeredness and dementia. These publications form the basis for the following analysis. For all identified 1023 publications, full texts were obtained and imported into the reference management software EndNote. To identify publications that use the concept of autonomy, all full texts and other fields in EndNote were searched for the terms *autonomy/Autonomie* and *self-determination/Selbstbestimmung*. The search for terms in all full texts enabled us to identify potentially relevant publications, even if autonomy was not the focus of a publication and therefore was not mentioned in the title, abstract or keywords. This search revealed 364 potentially relevant publications for which a full text screening was conducted by two researchers (JSK and JD); ultimately (following an update of the original search), 141 publications were included in this review. The inclusion criterion was that in the publication, the terms autonomy or self-determination were used with regard to persons with dementia. The exclusion criteria were that these terms were used in relation to other groups (e.g., professional care workers or family carers) or in other contexts. The flow of publications is displayed in Fig. 1, and the included publications are shown in Table S2 (this table can be found in an additional file (see Table S2)).

### Analysis

To analyse the included studies, we used an approach inspired by the thematic synthesis of Thomas and Harden [21]. As a first step, two researchers (JSK and JD) independently coded the relevant text passages in vivo and developed descriptive themes according to these codings. Then, the codes, codings and themes were compared, and differences were discussed to develop common codes and common descriptive themes. Building on these descriptive themes, the two researchers developed more abstract analytical themes together. This development contained several iterative loops in which codes and themes were renamed and regrouped until preliminary analytical themes were generated. These themes were discussed with the third author (MR) until the final analytical themes were established. The analysis revealed five analytical themes related to autonomy: “losing autonomy”, “assisting autonomy”, “stigma and autonomy”, “relevance of autonomy” and “relationship-based autonomy”. All analytical steps were performed using MAXQDA 2018. We have consciously opted for an integrative model, which makes the allocation within each theme. An example of the generation of in vivo codes and analytical themes is illustrated in Table 1. After constructing the analytical themes, we counted the number of the underlying text passages for each theme to quantify the different aspects of conceptual considerations. The systematic thematic assignment and frequency distribution of different interpretations offer the opportunity to determine areas in which much has already been achieved and those in which further research is needed.

**Table 1:** Illustration of the analytical steps

Original text passage	In vivo code	Descriptive theme	Analytical theme
“Rather, a person with dementia experiences loss of autonomy and personhood as a consequence of the relational behaviour of significant others (Sabat & Harre, 1992). For example, within an institution, the adapted self of a person with a dual disability can be either resident or inmate in custody. The role depends on the environment and its amenability to support existing roles or create opportunity for new ones (Sabat & Harre, 1992).”	“relational behaviour of others > loss of autonomy and personhood”	“behaviour of others leads to loss of autonomy of plwd”	“stigma and autonomy”

Since the analysis of the literature was guided by the goal of conceptually distinguishing how personhood is constituted with reference to the autonomy of people with dementia, we grouped these analytical themes under two different perspectives on personhood. Among the ways in which the concept of autonomy was discussed in the literature, it was indeed possible to find assertions that could be assigned to a rationalistic and cognitive concept of personhood as well as assertions that focused on a relational perspective to argue from an interactionist or social constructionist position.

## Findings

The following section provides an overview of the inductively obtained analytical themes regarding the concept of autonomy and their assignment to the distinct conceptions of personhood. Within each analytical theme, the first paragraph exemplifies the assignment to the respective conception of personhood. In order to clarify the contents of the analytical themes, an example is provided, which shows in which context the concept of autonomy was discussed by the respective authors. The detailed analysis describing the content of each analytical theme can be found in the appendix (see Table S3 in the Appendix). Important for the conceptual analysis is the distribution of incidence with regard to individual allocations. The first analytical theme, “losing autonomy”, is derived from the content of 50 passages of the included articles. The second analytical theme, “assisting autonomy”, is based on a total of 367 text passages. Thirty-nine text passages contributed to our understanding of the third analytical theme, “stigma and autonomy”. A total of 74 text passages formed the analytical theme “relevance of autonomy”, and the last analytical theme, “relationship-based autonomy”, was based on 13 text passages. The analysis shows that the analytical theme “assisting autonomy” is the one derived from the most text passages of included references.

### Analytical theme “losing autonomy”

In this analytical theme, the main argument is that dementia leads to a loss of autonomy, and this central idea is discussed in several ways in the literature. This analytical theme can be assigned to a rationalistic and cognitive concept of personhood because here, autonomy is seen mostly as an absolute value that is threatened by the degenerative processes of cognitive capacities caused by dementia.

For example, DeWaal discusses the loss of autonomy and control which is linked to diminished capacities of the rational individual [22]. In the course of the disease it gets more and more difficult to be and act autonomously as “…it gets more and more difficult properly to process information in a way that enables someone to make decisions and as a result the capacity to make informed judgements becomes impaired and carrying out complicated tasks takes more effort until later in the course of the disorder it becomes well-nigh impossible” [22]. Another example can be found in an article by Ames who discusses a “thin” understanding of personhood which is defined through functional criteria (e.g. rationality and autonomy) under the impact of a culture of extreme individualism [23]. Within this “thin” understanding it is stated, that these criteria “…are functionalist criteria and of course, if individuals lose the capacity to function, they no longer qualify for the attribution ‘person’” [23].

### Analytical theme “assisting autonomy”

In this analytical theme, the main argument is that dementia is related to a loss of (cognitive) capacities but that fading capacities can be assisted and supported by others.

In this discourse, we found assumptions of a process of decline in which– as cognitive abilities erode– being autonomous becomes increasingly difficult. The difference from the first analytical theme is the strong emphasis on caring so that the remaining capacities can be assisted by others. The basic assumption is that cognitive decline is caused by the course of the disease. However, authors focus on the opportunity, even if the progression of cognitive decline is unsustainable, for others to assist people living with dementia in re-establishing states that could be called partial autonomy. Nevertheless, because the fundamental assumption is that autonomy is lost, this analytical theme could also be assigned to a rationalistic and cognitive concept of personhood, although it refers to relational aspects in terms of how assistance can be provided.

As an example on how to assist autonomy, we found statements that focusing on the remaining capacities is necessary to support autonomy for people living with dementia. While discussing maintaining selfhood for people living with dementia, Manji points out that “…keeping autonomy at the forefront of practice, despite the profound losses associated with dementia,… recognize that the self remains intact until death and that the person, if supported, can make choice” [24]. Morhardt and Spira mention the assistance of autonomy when describing that “the personhood movement encourages those who interact with people with dementia to have an appreciation for the self-determination and empowerment of individuals to direct their own care through the expression of their wishes and objectives” [25]. And Vernooij-Dassen et al. [18] points out that the assistance of people living with dementia to use their capacities can be realized through an assessment of person-environment fit, person-directed goal setting and an interdisciplinary team support to maintain autonomy.

### Analytical theme “autonomy and stigma”

In this analytical theme, the main argument is that being autonomous is impeded by others through stereotyping and prejudgments. This analytical theme can be assigned to the concept of personhood and thus represents a relational perspective. The chosen term stigma refers to the idea of the social process of labelling, which in sociology is prominently represented by the work of Erving Goffman [26]. These processes of labelling have tremendous effects on direct interactions and the reciprocal constitution of personhood and could also have an impact on the societal attitude towards dementia on a macrolevel.

In the analytical theme “autonomy and stigma”, the point is not how to assist the remaining capacities or establish partial autonomy, as in this case, it is not a question of cognitive decline that hinders people from living a self-determined life. Rather, the problem is that people living with dementia are deprived of still existing ways of autonomy through the social environment. It is assumed that stigmatization processes impede people living with dementia from being autonomous, which is discussed in different ways in the literature. In accordance with Kitwood [6], we differentiate statements that either lead to malignant social perception or malignant social interaction. The first (malignant social perception) stigmatizes people living with dementia by predominantly perceiving aspects of the disease. The second (malignant social interaction) relates not only to one’s perception but also to one’s social interaction by initiating directed action that predominantly focuses on aspects of the disease. We also found examples of reactions of people living with dementia to these social stigmatization processes.

An example regarding the negative social perception of people living with dementia is found in an article by Hennelly and colleagues who refer to a statement from Kitwood by emphasizing that “…much of dementia care is oppressive because it is not based on the mutual values of trust, respect, and communication within caring relationships, leading to a diminution of autonomy, agency, and capacity among people with dementia” [27]. And, for instance, Heggestad and colleagues point towards the possibility of the dementia diagnosis becoming the “master status” of a person and state that “to be seen and treated as a diagnosis is the same as objectifying the person and is a threat to a person’s dignity” [28].

### Analytical theme “relevance of autonomy”

In this analytical theme, valuing autonomy is seen as contingent and variable, especially in the context of dementia. We assigned this theme to the relational perspective on personhood because it takes a constructionist position. Autonomy is not seen as an absolute value. Moreover, autonomy depends on individual preferences that have an impact on how a person might describe him- or herself or how he or she is seen by other members of society; therefore, the focus is mainly on the social processes that emerge from such a description.

In this analytical theme, the question arises of whether autonomy is generally important in the context of dementia. On the one hand, various authors highlight the importance of autonomy from an individualistic point of view, especially in the case of vulnerability. Other authors, however, explicate a shift of relevance and find that other values or aspects are more important for people living with dementia. In addition to these discourse lines of importance and unimportance, several authors discuss the contingency of the concept at both the individual and societal levels.

For example, Fazio and colleagues discuss literature reviews on person-centred care and state that there is a growing interest in autonomy, since “…there is a shift in focus away from the traditional biomedical model in favour of embracing personal choice and autonomy” [29]. In a study of Lopez and colleagues, the perspective of staff and family members of people living with dementia is highlighted, summarizing that rather than individual choice and autonomy that were “…endorsed by much of the person-centered literature, participants in this study valued connections with NH staff and other family members” [30].

### Analytical theme “relationship-based autonomy”

In this analytical theme, the rationalistic and individualistic concept of autonomy is rejected in favour of a relationship-based model and a social constructionist perspective on personhood.

In the literature, we found a perspective on dementia and autonomy that is closely related to the aforementioned analytical theme relevance. Here, the relational aspect is much more explicit and is not described only as a question of personal preference. Basically, the individualized concept of autonomy is challenged insofar as the decisions and actions of individuals are seen as always being affected by their (social) environment. Thus, strictly speaking, the idea of a rationally acting and freely deciding individual is rejected in favour of the assumption of a relationally embedded agent who decides depending on the influence of others. The identified statements refer to this concept of relational autonomy to different degrees. We assigned this analytical theme as the prime example to the relational and social constructionist perspective on personhood.

As an example Gilmour and Brannelly, discuss the perspective of citizenship and emphasize the critique on the idea of autonomy within that concept and “…in particular the idea that individuals make decisions in isolation from their social networks” [31]. Hughes claims a different concept of the person by outlining philosophical issues in dementia, and recommends “…that people with dementia must be seen in a context and considered in terms of situated agency: in other words, we are all dependent beings and our selfhood is not solely determined by what goes on in our heads” [32].

## Discussion

The analysis of the texts located in the literature regarding autonomy and dementia in the context of person-centred care revealed different understandings that were summed up under five different analytical themes. These analytical themes can be understood from two different perspectives on personhood.

In the first analytical theme, “losing autonomy”, the common ground is the assumption of a loss of autonomy, which is due to a loss of (mostly cognitive) capacities throughout the progression of dementia. The second analytical theme thematises the possibility of compensating for the presumed loss of capacities through the assistance of others (significant others, such as family carers or professional carers). In the third theme, the focus is no longer on fading capacities as dementia progresses. Autonomy is thematised in terms of stigma imposed by others, which results in (negative) positioning that impedes autonomous action. For the fourth analytical theme, an idea of contingency is paramount. Autonomy is not seen as a rigid construct that a person can win or lose or must strive for. There is a fundamental possibility of replacing and overriding it, as an intellectual or theoretical idea, with other values that might be more important for the subject living with dementia. The contradictory idea of emphasizing the value of autonomy in the case of vulnerability also appears here. In the final analytical theme, “relationship-based autonomy”, the idea of a rationally acting, freely deciding autonomous individual must be rejected in favour of a social constructionist conception of personhood. If one thinks about autonomy and dementia in this way, the idea of autonomy shifts towards a relational understanding that emphasises the social influences and processes that determine the decisions and actions of the subject.

As stated in the methods section of this article, the analysis was guided by the conceptual distinction of two perspectives on personhood. In examining the underlying concepts of each analytical theme more closely, we observed the possibility of assigning the different themes to these two perspectives. The first two themes have a common ground in the assumed loss of capacities as a direct impact of the dementia process, which results in the subject’s incapability to act autonomously. For the first two themes, we therefore implicitly supposed an individualistic and rationalistic concept of personhood, as the fundamental assumption is that of a rational individual who gains or loses the (mostly cognitive) abilities to make decisions and evaluate possible consequences. The third to fifth analytical themes are based on different fundamental assumptions. As autonomy in this conception is more an effect of interpersonal processes than an individual characteristic, the idea of the rational actor is conceptually no longer necessary. For these themes, we therefore supposed a relational concept of personhood because how personhood is socially constructed depends on the reciprocal and relational processes between at least two subjects and not on being an autonomous individual by retaining cognitive capacities.

To differentiate the perspectives on autonomy and dementia, it is helpful to focus on the underlying concept of personhood. It appears that the concept of autonomy either is constitutive of the concept of personhood or does not play a pivotal role in being a person at all. The results of the study clearly indicate that how to understand the role of the concept of autonomy in the understanding of personhood in dementia depends on the perspective, but different perspectives may have different consequences for communicating and dealing with people living with dementia. While the first perspective focuses mostly on deficits (and support for the person with dementia) caused by the disease that may hinder social participation or lead to social exclusion, the second perspective makes it possible to focus on the person living with dementia and the social processes that affect their personhood by not (over)emphasising the effects of dementia as a disease.

### Assistance– an example of a “caring paradox”?

Most of the included articles focus on the question of how to assist people living with dementia in managing their lost capacities. The reason for this emphasis might be that the topic of dementia is associated mostly with the topic of caring. Following the interpretation in this study, these articles therefore have a rational and individualistic understanding of personhood, as the fundamental assumption is that of a (cognitive) decline throughout the disease trajectory. However, the idea of assistance is not always easy and clearly delimitable. In this case, the central foundation is the loss of capacities, and the argumentation is mostly deficit- and/or problem-oriented. At the same time, social aspects, such as how to assist and who are the actors of caring, become paramount and open up the perspective towards relational aspects of the caring relationship.

The circumstance that the topic of assistance is the one that is most discussed in the literature also reveals a general problem in dementia care. If the idea of assistance is mostly deficit- or problem-oriented, it seems impossible to extend it beyond an underlying rationalistic perspective. By highlighting the aspects of the disease with the normative goal of improvement, it might contribute to the process of depersonalization, as mentioned by Kitwood [6]. The situation might be different if it were possible to open up the perspective towards relational aspects. In any case, it is important to obtain a better understanding of the social factors and processes that constitute the caring relationship. What becomes clear from these considerations is that the discussion of normality is essential here. Binary classifications of “healthy” or “ill” and the claim and goal of care to improve the health status of a person might reach their limits in the context of dementia. Additionally it should be noted that in nursing care, a continuum from being healthy to being ill is also discussed [33, 34]. Still, the idea of improvement in its very sense always requires a contrasting negative status. However, in the field of dementia such thinking of improvement becomes incomparably more complex when the status as a person is linked to cognitive abilities. What follows is that the claim of the idea of care can be questioned and thought of no longer mainly in terms of treatment and cure but rather in terms of relationships. The development of the expert standard “Relationship management in the care of people with dementia” [35] in Germany can be cited here as a fundamental step. By focusing on how relationships are formed, a paradigm shift has occurred in the care of people living with dementia, the opportunities of which lie in non-deficit- and resource-oriented care and thus in the recognition of people living with dementia as persons. At the same time, the challenges are assumed to be in the scientific examination of the underlying social processes and relationship structures, among other aspects.

What has become very clear throughout the analysis, and especially considering the idea of assisted autonomy, is the heterogeneity of perspectives on the concept of autonomy with regard to dementia. Depending on the perspective, different consequences arise for the underlying conception of personhood both in dealing with people living with dementia in direct interaction and in the societal image of and attitude towards them. The debate on autonomy in the context of dementia is not purely academic but has tangible consequences. This observation substantiates the relevance of applying different perspectives on autonomy to practical fields of dementia research and care where the concept is taken into consideration.

### Example of application: autonomy in the context of social health

As mentioned above, the concept of autonomy is quite important for the concept of social health, specifically in the context of dementia. It may then make sense to take the discussion on the operationalisation of social health as an example in applying different perspectives on autonomy and dementia to explicate different consequences and prerequisites for action options. To do so, we selected the second dimension, “managing life with some degree of independence”, operationalised by the INTERDEM taskforce on social health, and contextualised it within the ideas on autonomy related to the analytical themes found in the literature.

The INTERDEM taskforce suggested that this dimension (“managing life with some degree of independence”) could be realised if people living with dementia preserve their autonomy, are able to solve problems in daily life and are able to adapt to and cope with the consequences of the disease [16]. With reference to the first analytical theme, “losing autonomy”, it could be claimed that from a certain point, people living with dementia are unable to fulfil the requirements of this dimension due to the cognitive decline caused by the disease. Similarly, under the analytical theme “assisting autonomy”, it could be claimed that people living with dementia are increasingly unable to meet the requirements. Due to their declining capacities, they must be assisted and supported by others. A completely different view arises from the analytical theme “stigma and autonomy”. From this point of view, it could be stated that people living with dementia may well be in a position to meet the required conditions. However, this possibility is made more difficult for them because their actual existing capacity is denied by others based on judgements concerning the disease. As within the analytical theme “relevance of autonomy”, the relevance of the concept of autonomy in the context of dementia is fundamentally questioned and meeting the conditions of the second dimension may also lose relevance. Other values or conditions may therefore be more important than preserving autonomy in the context of dementia. The last analytical theme, “relationship-based autonomy”, may shift towards a research focus on the analysis of the processes that lead to the social construction and constitution of the specific content of a relevant dimension.

Thus far, the discussion has revealed two crucial points. On the one hand, it is clear that the perspective taken on personhood plays a pivotal role in how to approach and deal with people living with dementia. This is highly relevant for care, where it might be necessary to overcome a normative understanding of improvement by focusing on the reciprocal processes that constitute personhood and the caring relationship. Additionally, with regard to more practice-oriented research, it might be helpful to consider different conceptual perspectives while operationalising concepts such as social health towards possible psychosocial interventions. Furthermore, explicating implicitly used concepts might be helpful in changing practice, specifically since a change in attitude towards people living with dementia can enhance sensitivity in practice toward people living with dementia.

All in all, we want to emphasize the impact that a thematic synthesis– understood as part of a conceptual analyses– has for research. Dementia research benefits from such groundwork when thinking for example about the development of personalized intervention or while implementing participatory methods in dementia research or, beyond that, a broader debate on the methodological issues that follow from these perspectives.

### Limitations

Our findings refer to a specific area, by exploring and differentiating the concept of autonomy within the literature on person-centred dementia research and care. We chose this area because we expected that the analysis of the concept of autonomy in the context of dementia expand the debate on personhood and dementia and thus open up the opportunity for an elaborated understanding of personhood within the context of person-centredness and dementia. Nevertheless, it seems to be possible that the design of our review and the narrowing of our research question on the topic of autonomy within person-centred dementia research and care leads to a loss of relevant topics in critical gerontology that are associated with autonomy and dementia in general. Some could expect, that they find articles in our literature sample which for example highlight a critical cultural perspective or promoting the topic of citizenship within the context of dementia. These highly relevant topics have only been touched on in passing here due to the containment of the search strategy and design of our review. But also from a conceptual point of view there is a restriction as there seems to be a homogeneous understanding of autonomy and personhood is seen as paramount and a diversity perspective is only in the beginning. Leaving the specific frame of person-centred research and care and broadening the search strategy for the terms autonomy and dementia only, it might offer some relevant perspectives and topics in the literature which otherwise cannot be found.

The differentiation of the concept of autonomy within the literature on person-centred dementia research and care presented in this article should not be understood as a matured structural or analytical model. Although it could be a worthwhile idea to elaborate the conceptual exploration towards a practically relevant tool, we so far could only present preliminary impact. Basically, our review provides a descriptive approach, aiming to understand how far the exploration of the concept of autonomy within person-centred dementia research and care helps to concretize understandings of personhood in that context. Regarding our methodology, it is to say that we followed overall an interpretative approach for constructing the analytical themes. The descriptive statements were collected, interpretatively assigned and served to describe the respective analytical theme in more detail.

## Conclusion and prospect

The aim of our article was to conduct a conceptual analysis of the term autonomy in the context of person-centred dementia care. In the examination of the literature identified on this topic, it became clear that autonomy is used heterogeneously in the context of dementia. In-depth analysis indicated that these different understandings of autonomy could be assigned to two perspectives on people living with dementia. Considering these results against the background of the intention of a conceptual analysis, it becomes fundamentally clear that how people with dementia are perceived and recognized as persons depends on the underlying perspective on personhood. Depending on the perspective taken, there are different consequences for dementia care and dementia care research.

Since the work of Kitwood, the concept that people living with dementia are depersonalised as the disease is made paramount and the person behind the disease is negated has often been criticised. The conceptual analysis on autonomy and dementia opens up perspectives to either focus on the disease and the accompanying (cognitive) deficits or to see the person in the context of various social relations and situations. This distinction might be helpful in face-to-face situations in care, where carers sometimes must change their perspective and in regard to society and the awareness and acceptance of vulnerable groups. Regarding the context of social relations there are other aspects that appear to be important in the context of dementia care, but which are not covered by our review. There seems to be a need to also focus on citizenship and other identity related perspectives (such as being a member of a minority ethnic group, sex and gender or social class) which have not been part of our search strategy. Moreover, it prospectively seems important to work out how the elaborated analytical themes relate to the subjective and autobiographical and identity related perspectives of the people living with dementia themselves.

The application of heterogeneous perspectives on autonomy within the discussion on the operationalisation of social health in the context of dementia reveals different options and requirements for action. Here, the question arises of how these perspectives can be put into practice. Further research in this field may open up different perspectives that may then help to concretise operationalisation with regard to the possible development of future psychosocial interventions. To accomplish this, it might be helpful to further investigate the connection of the analytical themes differentiated in this article with the domains that emerged from the operationalisation of the concept of social health.

With regard to the initial question of how the concept of autonomy is discussed within the literature, the results presented in this article show that a relational perspective still seems to be in the minority compared to a rationalistic concept. Nevertheless, the discussion of the results also showed that a perspective that focuses mostly on deficits (such as fading cognitive capacities) may lead to social exclusion. The question then arises of whether this is truly an approach that pursues dealing with personhood under the signs of dementia or whether it falls short in addressing the complexity of the phenomenon. The explication of dementia as a cognitive problem has come a long way, but the distribution and assignments in the conceptual analysis show that there is nevertheless a need for further research on the supposed counterpart of relationality. If it is necessary to constitute personhood in ways other than cognition, then the debate in the context of dementia must shift towards a relational and social constructionist perspective on personhood as a reciprocal and mutual assignment. The relational perspective on personhood and dementia is far from self-explanatory. Rather, it justifies a desideratum for research and requires a deeper understanding and therefore further investigation.

### Electronic supplementary material

Below is the link to the electronic supplementary material.

Supplementary Material 1

Supplementary Material 2

Supplementary Material 3

## Data Availability

The datasets supporting the conclusions of this article are included within the article and its additional files.
