# Supplementary material for: Autonomy and its relevance for the construction of personhood in dementia– a thematic synthesis
Source: BMC Geriatr. 2024 Mar 14;24:255. doi: 10.1186/s12877-024-04808-6 (PMC10941450; doi:10.1186/s12877-024-04808-6)
Supplement: Supplementary file 1 — Supplementary Material 1 [file 12877_2024_4808_MOESM1_ESM.docx]

**Appendix 1: Methodological description of the literature review on person-centeredness and dementia and Table S1**

To answer the question of what is meant by the concept of person-centeredness and personhood in the context of dementia, a systematic literature search was conducted and applied in nine literature databases and metadatabases (MEDLINE, CINAHL, Scopus, PubPsych, Cochrane Library, sowiport, PsychInfo, Web of Science, and EBSCO-Geisteswissenschaften). In all databases, the terms dementia and person-centeredness and their synonyms were used to search titles, abstracts and keywords. An example of this search strategy for MEDLINE is given in Table 1. No time limits were set. A first search was conducted in November 2014, an update of the search was performed in July 2017 and in July 2022.

Table S1: Example of search strategy for MEDLINE via PubMed

| (dement*[TIAB] OR demenz[TIAB] OR alzheim*[TIAB] OR dementia[Mesh] OR “alzheimer disease”[Mesh]) AND (person-cent*[TIAB] OR Person-orient*[TIAB] OR client-cent*[TIAB] OR Client-orient*[TIAB] OR resident-cent*[TIAB] OR Resident-orient*[TIAB] OR patient-cent*[TIAB] OR Patient-orient*[TIAB]) |
| --- |
|  |

After duplicates were removed, the titles and abstracts of all identified publications were screened by two independent researchers (JSK and JD) against the predefined inclusion criteria: 1. the publication had to be written in English or German, and 2. the publication had to focus on person-centeredness and dementia. The date of publication, publication type or study design were not inclusion or exclusion criteria. Disagreements about inclusion were discussed until consensus was achieved. At the end of the screening of titles and abstracts, 1023 publications were included as potentially relevant for the scoping study. Because the search strategy was very sensitive and the inclusion criteria were wide, all relevant publications regarding person-centeredness and dementia were included at the end of this step.
