# Supplementary material for: Autonomy and its relevance for the construction of personhood in dementia– a thematic synthesis
Source: BMC Geriatr. 2024 Mar 14;24:255. doi: 10.1186/s12877-024-04808-6 (PMC10941450; doi:10.1186/s12877-024-04808-6)
Supplement: Supplementary file 2 — Supplementary Material 2 [file 12877_2024_4808_MOESM2_ESM.docx]

***Appendix 2: Table S2***

In the following table, all included publications are displayed.

| **Author** | **Aim** | **Publication/Study type** |
| --- | --- | --- |
| Aasgaard et al. (1) | to describe staff’s experiences of providing care to home-dwelling dementia clients through “home health care” services | - qualitative study - focus group interviews with eight registered nurses; phenomenological-hermeneutic analysis |
| Ahessy (2) | to highlight the role of person-centred music therapy in the care of people with Alzheimer's disease | - case study with one person with dementia |
| Ames (3) | to assess competing understandings of the person, to see whether there is a preferred understanding and to see whether this preferred understanding helps develop our understanding of what happens to the person with dementia | - scientific essay |
| Aubrecht and Keefe (4) | to examine how assumptions about dementia mediate social understandings of the nursing home and organize relations between the people who live in it and to analyse how dementia is imagined as an opportunity to affirm the legitimacy of social orders that privilege capacity and frame the meaning of personhood from the perspective of the “rational subject” | - secondary analysis of interviews |
| Barbosa et al. (5) | to assess the effects of a psychoeducational intervention, designed to improve direct care workers’ stress, burnout and job satisfaction, and person-centred communicative behaviour in people with dementia | - quantitative study - experimental study with a pre-post-test control group design, 53 direct care workers participating in the study |
| Beerens et al. (6) | to identify the degree of association between mood, activity engagement, activity location, and social interaction during everyday life of people with dementia living in long-term care facilities | - quantitative study - observational study - 115 persons with dementia |
| Behuniak (7) | to criticise the biomedical, social constructionist, and phenomenological models of dementia and to develop a political model of dementia | - scientific essay - using Rollo May’s theory of power |
| Bentwich et al. (8) | to explore possible differences among formal caretakers from varied cultural backgrounds in their perception of the autonomy and the human dignity of patients with dementia | - mixed-methods: semistructured interviews (content analysis) and questionnaires (regression analysis) - 197 professional carers (e.g., nurses and nurses` aides) |
| Bergland et al. (9) | to evaluate the psychometric properties of the Norwegian version of the Person-centred Climate Questionnaire – Staff version | - quantitative study - cross-sectional - 209 professional carers |
| Boumans et al. (10) | to understand *how* caregiving approaches and physical environment, including technologies contribute to the maintenance of autonomy and informal care provision for this population | - Literature review of peer-reviewed articles - 49 articles included - Realist logic of analysis was used, involving context, mechanism and outcome configurations |
| Boumans et al. (11) | to explore how residential care facilities (RCF) staff can enhance autonomy and improve informal care by looking at the influence of interactions (contact and approachability between residents, staff members and informal caregivers) and the physical environment, including the use of technologies | - realist evaluation multiple-case study - using document analyses, eight semi-structured interviews with staff members and relatives and 56 hours of observations of residents across two RCFs - realist logic of analysis was performed, involving Context-Mechanism-Outcome configurations |
| Brown Wilson et al. (12) | to develop, deliver, and evaluate a training program in care homes based on the principles of relationship-centred care | - mixed methods: quantitative questionnaires (descriptive statistics) and qualitative thematic content analysis - 11 professional carers, 6 persons with dementia, 4 family members, 3 managers |
| Bryden (13) | to consider a person-oriented approach to counselling, psychotherapy and rehabilitation for people with a diagnosis of dementia | - scientific essay |
| Buron (14) | to propose a Personhood Model for Dementia Care that is based on three identified levels of personhood | - scientific essay |
| Castillo (15) | to reveal the impact that cultural assumptions about dementia have on the care provided and to suggest a model of personhood-centred care | - scientific essay |
| Cayton (16) | to consider the role of narrative in people’s lives | - essay |
| Chapman et al. (17) | to explore the ways in which various modalities of the “self” are displayed in persons with dementia | - ethnographic observation of residents, family members, and care staff of an aged care facility - scientific essay |
| Chaudhury et al. (18) | to examine the role of the physical environment of dining rooms on positive outcomes of the dining experience of persons with dementia in long-term care facilities | - systematic literature review |
| Chenoweth et al. (19) | to evaluate the efficacy and cost-effectiveness of a person-centred approach to nursing care practices (PCC) and modifications to physical environment (PCE) in residential dementia care services | - quantitative study - cluster RCT - 38 care homes - 605 persons with dementia - 380 professional carers |
| Cherry et al. (20) | to describe a model of dementia care | - scientific essay |
| Chung (21) | to report on how coresident carers enhance their relatives’ autonomy and sense of self despite a progressive decline in cognitive function | - qualitative study - secondary analysis - qualitative interviews with family carers (individual interviews (n=15) and focus groups (n=21)) |
| Chung et al. (22) | to describe how family carers, through the caregiving journey, reaffirm and promote the agency of people with dementia | - qualitative study - secondary analysis - qualitative interviews with family carers (individual interviews (n=15) and focus groups (n=21) |
| Clarke et al. (23) | to explore aspects of providing information for people with dementia | - scientific essay |
| Clarke (24) | to consider challenges to mental health in older age groups and particularly the phenomenon of dementia | - scientific essay |
| Clark-McGhee and Castro (25) | to investigate how persons with dementia represent selfhood through words | - qualitative study - analysis of poems by 12 persons with dementia |
| Colomer and de Vries (26) | to identify perspectives and experiences of care assistants with PCC in nursing homes | - qualitative study - semistructured interviews with 13 care assistants |
| Cooney et al. (27) | to understand the perspectives of people with dementia, staff and relatives on reminiscence, its impact on their lives and their experience of care and caregiving | - qualitative study - grounded theory - in-depth interviews with 11 persons with dementia, 5 family members, 10 health care assistants, 9 registered nurses and 3 nurse managers |
| Cruz et al. (28) | to develop a multisensory and motor-based group activity program for residents with dementia and assess its impact on residents’ behaviour | - quantitative study - pilot study - analysis of behaviour of persons with dementia using video recordings collected during the sessions |
| de Waal (29) | to describe the loss of autonomy and control as the core problem in dementia and to highlight the individuality of the lived experience of dementia | - scientific essay |
| de Witt and Ploeg (30) | to describe health care professionals’ experiences of caring for older people with dementia living alone | - qualitative study - semistructured interviews with 15 professional carers |
| Dewing (31) | to introduce a person-centred approach to help nurses to maintain dignity and autonomy for people with dementia | - scientific essay |
| Dewing (32) | to critically review the traditional competency-based approach to informed consent in research with persons with dementia and to describe a new method based on person-centeredness to include persons with dementia in research | - scientific essay |
| Dewing (33) | to critically discuss approaches to person-centred care and the challenge of translating them into practice. | - scientific essay |
| Dewing (34) | to describe a process consent method for persons with dementia | - scientific essay |
| Dewing (35) | to explore central ideas around key theories of personhood, to critique Kitwood’s work on personhood, to summarize current critiques of Kitwood’s ideas and to provide a response that outlines why Kitwood’s ideas are still relevant | - scientific essay |
| Downs et al. (36) | to examine the key elements of a person-centred approach applied to those with severe dementia | - scientific essay |
| Downs (37) | to present the United Kingdom’s approach to person-centred care for persons with dementia | - scientific essay |
| Doyle (38) | 1. to examine how person-centred care is conceptualized by staff members; 2. to document interactions of direct care workers in a dementia care setting; 3. to assess the alignment of conceptualization of person-centred care and daily care interactions | - qualitative study - ethnography in a nursing home (dissertation) - interviews with 20 persons with dementia and 25 staff members |
| Doyle and Rubinstein (39) | to examine how person-centred care was defined, shaped, and practised by staff members within a dementia care setting | - qualitative study - ethnography in a nursing home (dissertation) - interviews with 20 persons with dementia and 25 staff members |
| Drayton et al. (40) | to describe how the multidisciplinary team implemented a variety of strategies to help staff intervene more effectively in meeting the care needs of a person suffering from several chronic diseases accompanied by dementia | - case description of one person with dementia in a hospital |
| Dupuis et al. (41) | to describe the Partnership in Dementia Care Alliance and the culture change process associated with this alliance | - scientific essay or case study |
| Dupuis et al. (42) | to describe the ‘authentic partnership’ approach and its relationship to the person-centred approach | - scientific essay or case study |
| Edvardsson et al. (43) | to describe the content of person-centred care as described by people with dementia, family members and staff in residential aged care | - qualitative study - focus groups or face-to-face interviews with 37 staff members, 11 persons with early-onset dementia, 7 persons with dementia, and 12 informal carers. |
| Edvardsson and Innes (44) | to present a critical comparative review of published tools measuring the person-centeredness of care for older people and people with dementia | - literature review |
| Edvardsson et al. (45) | to review the current knowledge about person-centred care for people with severe Alzheimer’s Disease and to highlight areas for further research | - literature review |
| Ericson et al. (46) | to understand the attributes of best care as defined by family and professional carers | - qualitative study - face-to-face interviews with 20 family carers and 17 professional carers |
| Estrada (47) | to present the voices of older adults living with several types of dementia, collected while working as a psychologist in a nursing home, in the first person to bring forward the person they are | - excerpts of interactions illustrate the basic psychological need of relatedness, which is built through interaction, stories, and touch, and the needs of competence and autonomy - the framework of this paper encompasses validation therapy, person-centered care, and self-determination theory - scientific essay |
| Evans et al. (48) | to evaluate the contribution that extra care housing can make to the long-term care and support of people with dementia | - mixed methods: quantitative research with 103 persons with dementia and 125 qualitative interviews with persons with dementia, their relatives, care staff, other tenants and managers from health and social care organizations. |
| Falk et al. (49) | to examine the effects and experiences of interinstitutional relocation on older persons' quality of life, well-being, and perceived person-centeredness | - mixed methods: quantitative questionnaires and semistructured interviews with 155 residents (112 diagnosed with dementia) |
| Fazio (50) | to review the origins and fundamentals of person-centred care, highlight innovations in thinking and practice, and discuss future opportunities | - literature review |
| Fazio (51) | to review the fundamental aspects of person-centred care and to discuss how they are essential in maintaining the self | - literature review |
| Fazio et al. (52) | to outline the history, components, and impact of person-centered care practices. | - literature review |
| Førsund et al. (53) | to interpret and synthesize knowledge regarding persons with dementia’s experience of space | - systematic meta-synthesis - 1386 articles, of which 136 were identified as eligible and were read and assessed using the CASP criteria - analysis inspired by qualitative content analyses. |
| Fox and Wilson (54) | to describe the experience of conducting person-centred advocacy for older people who have dementia | - case study |
| Frankowski and Clark (55) | to contribute to an understanding of how sexuality and intimacy are experienced within the social models of care providers in assisted living communities | - qualitative study - secondary analysis of three ethnographic studies |
| Gavan (56) | to determine whether a person-centred care or a recovery-based approach is more useful to dementia care | - literature review |
| Gilmour and Brannelly (57) | to trace conceptually shifts in health professional representations of people with dementia | - scientific essay |
| Gilpin (58) | to describe the Plantree model of patient-centred care and to examine its impact on caring for those with dementia | - essay |
| Gladman et al. (59) | to evaluate a specialist community-based dementia service | - qualitative study - ethnographic approach - focus groups with care staff - interviews with GPs, an old-age psychiatrist, NHS patient advocates, a representative of the Alzheimer’s Society and 15 persons with dementia |
| Godwin (60) | to examine whether assistive technology has a role in person-centred care and whether users can participate in a consultation process; furthermore, to specify the ethical considerations and benefits of assistive technology | - qualitative study - interviews with 9 persons with dementia, 9 family carers and 9 professional carers |
| Goodman at el. (61) | to explore how older people with dementia discuss their priorities and preferences for end-of-life care | - qualitative study - secondary analysis of 18 interviews with persons with dementia |
| Graf and Krebs-Roubicek (62) | to describe the practice of competence assessment in dementia in Switzerland | - essay |
| Gridley et al. (63) | to identify evidence about services for people with complex needs and to identify evidence of the (cost-)effectiveness of these services and research gaps | - systematic literature review (scoping study) |
| Gurland et al. (64) | to outline interprofessional strategies that could relieve restrictions or distortions of the choices and choosing processes imposed by ageing, ill health, or a restrictive environment | - scientific essay |
| Hajime et al. (65) | to analyse declines in different forms of ADL | - quantitative study - observational study - 39 persons with dementia and their carers |
| Han and Radel (66) | to explore the experience and impact of a person-centred social program for community- dwelling people in earlier stages of dementia | - qualitative study - phenomenological analysis - 5 persons with dementia |
| Heggestad et al. (67) | to gain more knowledge about how persons with dementia and their relatives experience their dignity being maintained or harmed in nursing homes | - qualitative study - phenomenological analysis - 15 persons with dementia and 7 relatives of the persons with dementia |
| Heggestad et al. (68) | to investigate how life in Norwegian nursing homes may affect experiences of dignity among persons with dementia | - qualitative study - phenomenological analysis - 15 persons with dementia |
| Helgesen et al. (69) | to explore patient participation in everyday activities for persons with dementia in special care units in nursing homes | - qualitative study - grounded theory - 8 persons with dementia - 17 professional carers |
| Hennelly et al. (70) | to present experiences and perceptions of people with dementia on personhood in dementia care | - qualitative evidence synthesis - thematic synthesis was conducted on 20 papers |
| Higgs and Gilleard (71) | to investigate the concept of personhood and its application to care practices for people with dementia | - scientific essay |
| Hilgeman et al. (72) | to test the Preserving Identity and Planning for Advance Care (PIPAC) intervention for individuals in the early stages of dementia | - quantitative study - RCT - 19 persons with dementia and their family carers |
| Hilton and Moniz-Cook (73) | to examine whether the personality dimensions of sociotropy and autonomy remain stable in older people with dementia | - quantitative study - observational study - 63 persons with dementia and 100 persons without dementia |
| Hirsch (74) | to describe socio and physiotherapy for persons with dementia | - scientific essay |
| Hoe and Thompson (75) | to provide an overview of factors relevant to dementia care today | - scientific essay |
| Hughes (76) | to consider the philosophical literature from the previous 18 months relevant to dementia | - literature review |
| Hughes (77) | to examine recent writings relevant to acquired cognitive impairment in an attempt to reveal some of the underlying conceptual issues | - literature review |
| Hughes et al. (78) | to consider the importance of the notion of centeredness in general and the reasons for the existence of different types of centeredness | - systematic literature review |
| Hughes and Beatty (79) | to examine the notion of personhood and to show how it offers a robust conceptual underpinning for person-centred care | - scientific essay |
| Hunter et al. (80) | to investigate the association of personal and organizational-environmental characteristics with self-reported person-centred behaviours in long-term residential care settings | - quantitative study - observational study - 108 professional carers and other employees |
| Irving and Lakeman (81) | to synthesize two approaches to care – mental health recovery and person-centred care – and apply them to early-stage dementia care | - scientific essay |
| Jablonski-Jaudon et al. (82) | to describe a personalized practice originally conceived as a way to prevent and minimize care-resistant behaviour to provide mouth care to older adults with dementia | - scientific essay |
| Janes et al. (83) | to understand the factors that influence the utilization of knowledge about person-centred care and the interrelationships among these factors and the use of knowledge about person-centred care in everyday practice in long-term care facilities | - qualitative study - grounded theory - 20 personal support workers |
| Jenkins et al. (84) | to discuss the creation of four theatrical vignettes about living with early-onset dementia | - qualitative study - drama workshop and focus group - 4 persons with dementia, 13 family members, 3 practitioners, 1 researcher, 2 workshop facilitators and 2 workshop actors |
| Jennings et al. (85) | to elicit patient-centred goals for dementia care | - qualitative study - focus groups - 6 persons with dementia, 36 family carers |
| Johnston et al. (86) | to assess the feasibility, acceptability and potential effectiveness of dignity therapy to improve quality of life and reduce psychological and spiritual distress in older people with early-stage dementia | - mixed methods - interviews, summaries, focus groups and standardized outcome measures - 7 persons with dementia, 7 family members, 7 stakeholder participants, 6 focus group members |
| Kaufmann and Engel (87) | to examine the empirical relevance of Kitwood’s model of psychological needs against the background of the subjective experiences of individuals with moderate or severe dementia and to elaborate this model in order to develop a conceptual framework of well-being of individuals with moderate or severe dementia. | - qualitative study - content analysis - interviews with 19 persons with dementia |
| Kirkley et al. (88) | to understand the impact of organizational culture on the provision of respite care or short breaks for people with Alzheimer’s disease and other dementias and their informal carers | - qualitative study - semistructured interviews and focus groups with 70 professionals |
| Kolanowski et al. (89) | to document how staff members in nursing homes obtain information needed to implement a person-centred approach to dementia care, particularly with respect to BPSD, and how they communicate this information to other staff. | - qualitative study - focus groups with 59 professional carers |
| Kreps (90) | to introduce ‘skeuomorphic reassurance’ as a guiding principle for human interfaces in technological design, particularly for older people and people with dementia. | - scientific essay |
| Lann-Wolcott et al. (91) | to assess the predictive and construct validity of the Global Behaviour Scale and the Person-Centred Behaviour Inventory | - quantitative study - observational study - 20 persons with dementia and 53 professional carers |
| Leverton et al. (92) | to investigate how homecare workers support or inhibit independence in people living with dementia | - ethnographic study - 100 h of participant observations with homecare workers (n=16) supporting people living with dementia (n=17) - 82 qualitative interviews with people living with dementia (n=11), family carers (n=22), homecare managers and support staff (n=11), homecare workers (n=19) and health and social care professionals (n=19) - triangulated data and analysed findings thematically |
| Li and Porock (93) | to synthesize current evidence of the effects of multiple person-centred care models on resident outcomes | - systematic literature review |
| Lopez et al. (94) | to understand family members’ perspectives on person- and family-centred end-of-life care provided to nursing home residents with advanced dementia | - qualitative study - thematic analysis - semistructured, open-ended interviews with 25 family members of persons with dementia |
| Manji (95) | to understand the implications of a specialized model of support within community living that has evolved to support individuals with a dual disability | - qualitative study - case study (dissertation) - 4 persons with dementia, 4 family members, 4 professional carers, 4 staff members |
| Manthorpe and Samsi (96) | 1) to chart the evolution of the concept of person-centred care to the policy of personalization, 2) to summarize emerging and conflicting evidence about the implications of personal budgets in England on older people with mental health problems such as dementia and their families | - scientific essay |
| Martin (97) | to explore the decision-making processes of persons with dementia | - scientific essay |
| Martinez et al. (98) | to review the procedures and instruments used in the assessment of services using the person-centred care approach | - systematic literature review |
| McAllister and Silverman (99) | to compare the experiences of persons with dementia of two different nursing homes with different care models | - qualitative study - ethnography with 16 persons with dementia |
| McClendon and Smyth (100) | 1) to expand the knowledge about the number and meanings of the dimensions of quality of care (QOC) received by persons with dementia at home, 2) to create valid and reliable measurement scales for these dimensions and to discover characteristics of family carers who provide better QOC on these dimensions and characteristics of persons with dementia who receive better QOC | - quantitative study - observational study - 148 family carers |
| McIntyre (101) | to offer an alternative to the image of the family caregiver as victim through an exploration of the lived experience of caring for a person with dementia in community | - scientific essay |
| Mitchell and Templeton (102) | to discuss ethical considerations of doll therapy for people with dementia | - scientific essay |
| Morhardt and Spira (103) | to discuss person-centred care and relationship-centred care | - scientific essay |
| Niemeijer et al. (104) | to explore how clients in residential care experience surveillance technology in order to assess how surveillance technology might influence autonomy | - qualitative study - ethnography in two nursing homes - formal interviews with 12 professional and 2 family carers |
| Nolan et al. (105) | 1) to critically examine the assumptions underpinning person-centeredness and to suggest that a relationship-centred approach to care might be more appropriate; 2) to describe the potential dimensions of relationship-centred care and to consider implications for further development | - scientific essay |
| Nowell et al. (106) | to understand the subjective experiences of personhood experienced by those with dementia | - qualitative study - phenomenological analysis - 7 persons with dementia |
| O'Dwyer (107) | to explore the development of minimum standards for residential care settings for older people in Ireland to address this gap in the understanding of person-centred care | - case study - discourse analyses - semistructured interviews with 32 members of an Irish agency |
| O'Sullivan (108) | to present the combination of approaches used to enable residents of two secure dementia units to live life within their ability and without antipsychotic drugs | - scientific essay |
| Palmer (109) | to report on the experiences of family caregivers who admitted their spouse or parent with probable Alzheimer’s disease to long-term care | - qualitative study - phenomenological analysis - 15 family carers |
| Parker (110) | to critically consider the conception of ‘personhood’ and to explore some of the philosophical challenges and practical difficulties raised by this debate in the context of care management in the UK | - scientific essay |
| Powers (111) | to develop a taxonomy of commonplace ethical issues, taking into account resident, family member, and nursing home staff member points of view. | - qualitative study - anthropological study combining observation in in-depth interviews - 30 persons with dementia, their family carers and professional carers |
| Reed and Tilly (112) | to summarize recommendations that offer guidance in ensuring the physical safety and personal autonomy of residents across 3 specific care areas: (1) resident wandering, (2) resident falls, and (3) physical restraint-free care | - essay |
| Robinson and Gallagher (113) | to describe the impact that culture change has on quality of life of nursing home residents and the change to person-centred care within nursing facilities | - scientific essay |
| Robinson et al. (114) | to develop and evaluate an educational intervention for old-age psychiatrists to promote patient-centred care in their consultations with people with dementia and their carers | - quantitative study - experimental study - 41 professional carers |
| Robinson et al. (115) | to explore the perspectives of different stakeholders in the management of wandering in dementia | - systematic review - qualitative study - 4 focus groups with 10 professional carers, 3 family carers and 6 persons with dementia |
| Rokstad et al. (116) | to investigate the psychometric properties of P-CAT in a sample of staff working in residential units for older people | - quantitative study - 753 professional carers |
| Rondon-Sulbaran et al. (117) | to present the experiences of formal carers working in technology-enriched supported accommodation for people living with dementia, examining their care-giving role from a person-centred care perspective | - qualitative study - 21 semi-structured interviews were conducted with formal carers - data were analysed following a thematic approach |
| Round et al. (118) | to understand the framework within which common instruments to measure quality of life have been developed, critique these instruments with respect to patients with severely restricted capacity and develop a new way of thinking about how to value health-related quality of life in such patients | - scientific essay |
| Rushton and Edvardsson (119) | to explore concepts of space and examine their implications for the delivery of care to older people who are cognitively impaired and to draw on how space has been constructed within the nursing literature that refers specifically to acute care | - scientific essay |
| Sauer et al. (120) | to compare a specific art program for persons with dementia with traditional art activities in regard to well-being and ill-being | - quantitative study - experimental study - 38 persons with dementia |
| Savundranayagam and Moore-Nielsen (121) | to investigate whether language-based strategies for effective communication with persons with dementia are person-centred | - qualitative study - audio-recorded conversations of 13 persons with dementia and their professional carers |
| Schwartz et al. (122) | to present the use of a patient-centred interdisciplinary process by applying core ethical principles to a case study involving nutrition support | - scientific essay |
| Sinclair et al. (123) | to understand how Australian health and legal professionals conceptualised their professional roles in the practice of providing decision-making support for people living with dementia | - qualitative study - In-depth, semi-structured interviews were conducted with 29 health and legal professionals involved in providing care or services for people with dementia - methods were informed by grounded theory principles |
| Smebye and Kirkevold (124) | to increase the understanding of the nature and quality of relationships between persons with dementia, family carers and professional caregivers and how these relationships influence personhood in people with dementia | - qualitative study - hermeneutic analyses - 10 persons with dementia - 10 family carers - 10 professional carers |
| Snoeren et al. (125) | to examine what contributes to the improvement of participation of older people with dementia in daily occupational and leisure activities | - qualitative study - action research/case study - 22 persons with dementia - 20 professional carers - 18 students |
| Snowdon (126) | to discuss recent findings and observations about psychogeriatric services in the community and in long-term care facilities and to suggest how to improve or develop such services | - literature review |
| Stechl et al. (127) | to report the main results of a study in which the perception and coping processes of persons with dementia were reconstructed | - qualitative study - interviews with 13 persons with dementia |
| Sugihara et al. (128) | to present a vision of person-centred care based on the use of information and communication technology to maintain autonomy and continuity in residents’ lives and to develop a road map to realizing assistive technologies | - scientific essay |
| Swinnen (129) | to study the techniques that enhance dialogue in a poetry intervention | - qualitative study - ethnographic analysis |
| Teng et al. (130) | to describe experiences and perspectives of people with dementia and their family caregivers in making decisions about institutional care placement | - literature review |
| Tetrault et al. (131) | to explore the view of people in the early stage of dementia on planning for future care | - qualitative interview study with a semistructured interview guide - data were analyzed according to the Qualitative Analysis Guide of Leuven |
| Thomsen et al. (132) | to point out the importance of person-centred care when caring for people with dementia | - scientific essay |
| Thornton (133) | to discuss person-centred care as an ethical approach to care for people with dementia living in long-term care residential settings | - scientific essay |
| Vernooij-Dassen et al. (134) | to introduce the notion of social health as applied to dementia care research | - guest editorial |
| Viau-Guay et al. (135) | to analyse caregivers’ assessment of the "relationship-based care" approach’s usefulness and their capacity, after training, to apply it to care practices | - quantitative study - secondary analyses - survey with open-ended questions - 392 professional carers |
| Watson (136) | to examine the role of embodied and interembodied selfhood within care-giving/care-receiving relationships in a specialist dementia care home | - ethnographic approach |
| White et al. (137) | to empirically test items of a measure designed to assess person-directed care practices in long-term care | - quantitative study - 430 professional carers |
| Wilberforce et al. (138) | to identify and synthesize the key service attributes that are considered person-centred | - systematic literature review |
| Williams et al. (139) | to examine whether a person-centred care program demonstrates positive outcomes | - mixed methods - quantitative: experimental pre-post design - qualitative: focus group and individual interviews |
| Williams et al. (140) | to test the feasibility and effects of automated digital displays of resident photographs to remind staff of resident personhood | - quantitative study - experimental study with a pre-post design - 6 persons with dementia - 10 professional carers |
| Wolfe et al. (141) | to explore the viewpoints of people with dementia and family carers regarding the meaning of autonomy with a view to informing rights-based practice | - Q-method investigation - Twenty participants, people living with dementia and family carers, each conducted a Q-sort of statements regarding the meaning of autonomy - by-person factor analysis was used to identify patterns |

**References**

1. Aasgaard HSF, Lisbeth; Landmark, Bjørg. Nurses’ Experiences of Providing Care to Dementia Patients Through Home Health Care: After Further Training and a Reorganization of Nursing Resources. Home Health Care Management & Practice. 2014;26(4):230-8.

2. Ahessy B. Song writing with clients who have dementia: A case study. Arts in Psychotherapy. 2017;55:23–31.

3. Ames S. What happens to the person with dementia? Journal of Religion, Spirituality & Aging. 2016;28(1-2):118–35.

4. Aubrecht K, Keefe J. “Everybody has different levels of why they are here”: Deconstructing domestication in the nursing home setting. In: Rembis M, editor. Disabling Domesticity. New York: Palgrave Macmillan US; 2016. p. 215–39.

5. Barbosa A, Nolan M, Sousa L, Marques A, Figueiredo D. Effects of a Psychoeducational Intervention for Direct Care Workers Caring for People With Dementia: results From a 6-Month Follow-Up Study. American journal of alzheimer's disease and other dementias [Internet]. 2016; 31(2):[144-55 pp.]. Available from: <http://onlinelibrary.wiley.com/o/cochrane/clcentral/articles/870/CN-01200870/frame.html>

<http://journals.sagepub.com/doi/pdf/10.1177/1533317515603500>.

6. Beerens HC, Zwakhalen SMG, Verbeek H, F EST, Jolani S, Downs M, et al. The relation between mood, activity, and interaction in long-term dementia care. Aging Ment Health. 2016:1-7.

7. Behuniak SM. Toward a political model of dementia: Power as compassionate care. Journal of Aging Studies. 2010;24(4):231-40.

8. Bentwich ME, Dickman N, Oberman A. Human dignity and autonomy in the care for patients with dementia: Differences among formal caretakers from various cultural backgrounds. Ethnicity & health. 2016:1–21.

9. Bergland AK, M.; Edvardsson, D. Psychometric properties of the Norwegian Person-centred Climate Questionnaire from a nursing home context. Scandinavian Journal of Caring Sciences. 2012;26(4):820-8.

10. Boumans J, Van Boekel LC, Baan CA, Luijkx KG, Heyn PC. How Can Autonomy Be Maintained and Informal Care Improved for People with Dementia Living in Residential Care Facilities: A Systematic Literature Review. Gerontologist. 2019;59(6):E709-E30.

11. Boumans J, van Boekel LC, Verbiest MEA, Baan CA, Luijkx KG. Exploring how residential care facilities can enhance the autonomy of people with dementia and improve informal care. Dementia. 2022;21(1):136-52.

12. Brown Wilson CS, C.; Pilling, M.; Keady, J. The senses in practice: Enhancing the quality of care for residents with dementia in care homes. Journal of Advanced Nursing. 2013;69(1):77-90.

13. Bryden C. A person-centred approach to counselling, psychotherapy and rehabilitation of people diagnosed with dementia in the early stages. Dementia (14713012). 2002;1(2):141-56.

14. Buron B. Levels of Personhood: A Model for Dementia Care. Geriatric Nursing. 2008;29(5):324-32.

15. Castillo EH. Doing dementia better: Anthropological insights. Clinics in Geriatric Medicine. 2011;27(2):273-89.

16. Cayton H. Telling stories: choices and challenges on the journey of dementia. Dementia (14713012). 2004;3(1):9-17.

17. Chapman M, Philip J, Komesaroff P. A person-centred problem. Hum Soc Sci Comm. 2022;9(1).

18. Chaudhury HH, L.; Badger, M. The role of physical environment in supporting person-centered dining in long-term care: A review of the literature. American Journal of Alzheimer's Disease and other Dementias. 2013;28(5):491-500.

19. Chenoweth LK, M.; Luscombe, G.; Forbes, I.; Jeon, Y. H.; Parbury, J. S.; Brodaty, H.; Fleming, R.; Haas, M. Study Protocol of a Randomised Controlled Group Trial of Client and Care Outcomes in the Residential Dementia Care Setting. Worldviews on Evidence-Based Nursing. 2011;8(3):153-65.

20. Cherry BC, K.; Waters, C.; Hawkins, W. W.; McGrew, P.; Satterwhite, L. J.; Stepien, J.; Ruppelt, W.; Herring, K. Social compatibility as a consideration in caring for nursing home residents with dementia. American Journal of Alzheimer's Disease and other Dementias. 2008;23(5):430-8.

21. Chung P. Professionals partnering with family carers in home-based activity for those with dementia. WFOT Bulletin. 2013;67:9-16.

22. Chung PYF, Ellis-Hill C, Coleman P. Supporting activity engagement by family carers at home: maintenance of agency and personhood in dementia. International journal of qualitative studies on health and well-being. 2017;12(1):1267316.

23. Clarke CLA, J.; Gibb, C. E. Information in dementia care: Sense making and a public health direction for the UK? International Journal of Older People Nursing. 2011;6(3):237-43.

24. Clarke J. Adverse factors and the mental health of older people: Implications for social policy and professional practice. Journal of Psychiatric and Mental Health Nursing. 2005;12(3):290-6.

25. Clark-McGhee K, Castro M. A narrative analysis of poetry written from the words of people given a diagnosis of dementia. Dementia. 2015;14(1):9-26.

26. Colomer J, de Vries J. Person-centred dementia care: a reality check in two nursing homes in Ireland. Dementia. 2016;15(5):1158-70.

27. Cooney AH, A.; Murphy, K.; Casey, D.; Devane, D.; Smyth, S.; Dempsey, L.; Murphy, E.; Jordan, F.; O'Shea, E. 'Seeing me through my memories': A grounded theory study on using reminiscence with people with dementia living in long-term care. Journal of Clinical Nursing. 2014.

28. Cruz JM, Alda; Barbosa, Ana; Figueiredo, Daniela; Sousa, Liliana X. Making sense(s) in dementia: A multisensory and motor-based group activity program. American Journal of Alzheimer's Disease and Other Dementias. 2013;28(2):137-46.

29. De Waal H. Rethinking dementia: How autonomy and control can be fostered through the development of person centred services. Working with Older People. 2014;18(2):82-9.

30. de Witt L, Ploeg J. Caring for older people living alone with dementia: Healthcare professionals’ experiences. Dementia. 2016;15(2):221-38.

31. Dewing J. Dementia. Part 1: Person-centred care. Professional nurse (London, England). 1999;14(8):585-8; quiz 3-4.

32. Dewing J. From ritual to relationship: A person-centred approach to consent in qualitative research with older people who have dementia. Dementia: The International Journal of Social Research and Practice. 2002;1(2):157-71.

33. Dewing J. Concerns relating to the application of frameworks to promote person-centredness in nursing with older people. Journal of Clinical Nursing. 2004;13(3 A):39-44.

34. Dewing J. Participatory research: a method for process consent with persons who have dementia. Dementia (14713012). 2007;6(1):11-25.

35. Dewing J. Personhood and dementia: Revisiting Tom Kitwood's ideas. International Journal of Older People Nursing. 2008;3(1):3-13.

36. Downs MS, Neil; Froggatt, Katherine. Person-centred Care for People with Severe Dementia. In: Burns AW, Bengt, editor. Severe dementia. New York, NY, US: John Wiley & Sons Ltd; 2006. p. 193-204.

37. Downs M. Putting People-and Compassion-First: The United Kingdom's Approach to Person-Centered Care for Individuals with Dementia. Generations. 2013;37(3):53-9.

38. Doyle PJ. Definitions, interpretations, and uses of person-centered care in a dementia-specific long-term care setting: A cultural analysis. US: ProQuest Information & Learning; 2012.

39. Doyle PJR, R. L. Person-Centered Dementia Care and the Cultural Matrix of Othering. Gerontologist. 2013.

40. Drayton SC, A.; Allen, C. Dilemmas in providing patient-focused care. CANNT journal = Journal ACITN. 2003;13(4):30-3.

41. Dupuis S, McAiney CA, Fortune D, Ploeg J, Witt LD. Theoretical foundations guiding culture change: The work of the Partnerships in Dementia Care Alliance. Dementia. 2016;15(1):85-105.

42. Dupuis SLG, Jennifer; Carson, Jennifer; Whyte, Colleen; Genoe, Rebecca; Loiselle, Lisa; Sadler, Leah. Moving beyond patient and client approaches: Mobilizing ‘authentic partnerships’ in dementia care, support and services. Dementia: The International Journal of Social Research and Practice. 2012;11(4):427-52.

43. Edvardsson DF, Deirdre; Nay, Rhonda. Promoting a continuation of self and normality: person-centred care as described by people with dementia, their family members and aged care staff. Journal of Clinical Nursing. 2010;19(17/18):2611-8.

44. Edvardsson DI, A. Measuring person-centered care: A critical comparative review of published tools. Gerontologist. 2010;50(6):834-46.

45. Edvardsson DW, B.; Sandman, P. Person-centred care of people with severe Alzheimer's disease: current status and ways forward. The Lancet Neurology. 2008;7(4):362-7.

46. Ericson IH, I.; Lundh, U.; Nolan, M. What constitutes good care for people with dementia? British journal of nursing (Mark Allen Publishing). 2001;10(11):710-4.

47. Estrada R. Listen and You Will See the Person Through the Dementia. J Pers Oriented Res. 2021;7(2):88-97.

48. Evans SF, Tina; Means, Robin; Vallelly, Sarah. Supporting independence for people with dementia in extra care housing. Dementia: The International Journal of Social Research and Practice. 2007;6(1):144-5.

49. Falk HW, H.; Persson, L. O. Frail Older Persons' Experiences of Interinstitutional Relocation. Geriatric Nursing. 2011;32(4):245-56.

50. Fazio S. Person-Centered Care in Residential Settings: Taking a Look Back While Continuing to Move Forward. Alzheimer's Care Today. 2008;9(2):155-61.

51. Fazio S. The enduring self in people with Alzheimer's: Getting to the heart of individualized care. Baltimore, MD, US: Health Professions Press; 2008.

52. Fazio S, Pace D, Flinner J, Kallmyer B. The Fundamentals of Person-Centered Care for Individuals with Dementia. Gerontologist. 2018;58:S10-S9.

53. Førsund LH, Grov EK, Helvik AS, Juvet LK, Skovdahl K, Eriksen S. The experience of lived space in persons with dementia: A systematic meta-synthesis. BMC Geriatr. 2018;18(1).

54. Fox MW, L. Person-centered advocacy for people with dementia... first of two articles. Journal of Dementia Care. 2007;15(2):17-9.

55. Frankowski ACC, L. J. Sexuality and Intimacy in Assisted Living: Residents' Perspectives and Experiences. Sex Res Soc Policy. 2009;6(4):25-37.

56. Gavan J. Exploring the usefulness of a recovery-based approach to dementia care nursing. Contemporary Nurse: A Journal for the Australian Nursing Profession. 2011;39(2):140-6.

57. Gilmour JAB, T. Representations of people with dementia - subaltern, person, citizen. Nursing Inquiry. 2010;17(3):240-7.

58. Gilpin L. The Planetree Model: Its impact on caring for those with dementia. Alzheimer's Care Quarterly. 2006;7(4):273-7.

59. Gladman JRFJ, R. G.; Radford, K.; Walker, E.; Rothera, I. Person-centred dementia services are feasible, but can they be sustained? Age and Ageing. 2007;36(2):171-6.

60. Godwin B. The ethical evaluation of assistive technology for practitioners: A checklist arising from a participatory study with people with dementia, family and professionals. Journal of Assistive Technologies. 2012;6(2):123-35.

61. Goodman CA, Sarah; Elmore, Natasha; Machen, Ina; Mathie, Elspeth. Preferences and priorities for ongoing and end-of-life care: A qualitative study of older people with dementia resident in care homes. International Journal of Nursing Studies. 2013;50(12):1639-47.

62. Graf MK-R, Eva. Practice of competence assessment in dementia: Switzerland. In: Stoppe G, editor. Competence assessment in dementia. New York, NY, US: Springer Publishing Co; 2008. p. 166-8.

63. Gridley KB, Jenni; Glendinning, Caroline. Good practice in social care for disabled adults and older people with severe and complex needs: Evidence from a scoping review. Health & Social Care in the Community. 2014;22(3):234-48.

64. Gurland BJG, R. V.; Mitty, E.; Toner, J. The choices, choosing model of quality of life: Clinical evaluation and intervention. Journal of Interprofessional Care. 2009;23(2):110-20.

65. Hajime TA, Kokuryu; Tomoko, Kubota; Hiroko, Yamada. Relative Preservation of Advanced Activities in Daily Living among Patients withMild-to-Moderate Dementia in the Community and Overview of Support Provided by Family Caregivers. International Journal of Alzheimer's Disease. 2012:1-7.

66. Han A, Radel J. The Benefits of a Person-Centered Social Program for Community-Dwelling People with Dementia: Interpretative Phenomenological Analysis. Activities, Adaptation & Aging. 2017;41(1):47–71.

67. Heggestad AKT, Nortvedt P, Slettebø Å. Dignity and care for people with dementia living in nursing homes. Dementia. 2015;14(6):825-41.

68. Heggestad AKTN, P.; Slettebo, A. 'Like a prison without bars': Dementia and experiences of dignity. Nursing Ethics. 2013;20(8):881-92.

69. Helgesen AKL, M.; Athlin, E. 'Patient participation' in everyday activities in special care units for persons with dementia in Norwegian nursing homes. International Journal of Older People Nursing. 2010;5(2):169-78.

70. Hennelly N, Cooney A, Houghton C, O'Shea E. Personhood and Dementia Care: A Qualitative Evidence Synthesis of the Perspectives of People with Dementia. Gerontologist. 2021;61(3):E85-E100.

71. Higgs P, Gilleard C. Interrogating personhood and dementia. Aging & Mental Health. 2016;20(8):773-80.

72. Hilgeman MMA, Rebecca S.; Snow, A. Lynn; Durkin, Daniel W.; DeCoster, Jamie; Burgio, LouisD. Preserving Identity and Planning for Advance Care (PIPAC): preliminary outcomes from a patient-centered intervention for individuals with mild dementia. Aging & Mental Health. 2014;18(4):411-24.

73. Hilton CM-C, Esme. Examining the personality dimensions of sociotropy and autonomy in older people with dementia: Their relevance to person centred care. Behavioural and Cognitive Psychotherapy. 2004;32(4):457-65.

74. Hirsch RD. Sozio- und Psychotherapie bei Alzheimerkranken. Zeitschrift für Gerontologie und Geriatrie. 2001;34(2):92-100.

75. Hoe JT, Rachel. Promoting positive approaches to dementia care in nursing. Nursing Standard. 2010;25(4):47-56.

76. Hughes JC. Philosophical issues in dementia. Current Opinion in Psychiatry. 2013;26(3):283-8.

77. Hughes JC. Conceptual issues in 'cognitive impairment'. Current Opinion in Psychiatry. 2015;28(2):188–93.

78. Hughes JCB, C.; May, C. Types of centredness in health care: themes and concepts. Medicine Health Care and Philosophy. 2008;11(4):455-63.

79. Hughes JCB, A. Understanding the person with dementia: A clinicophilosophical case discussion. Advances in Psychiatric Treatment. 2013;19(5):337-43.

80. Hunter PV, Hadjistavropoulos T, Thorpe L, Lix LM, Malloy DC. The influence of individual and organizational factors on person-centred dementia care. Aging & Mental Health. 2016;20(7):700-8.

81. Irving KL, R. Reconciling mental health recovery with screening and early intervention in dementia care. International Journal of Mental Health Nursing. 2010;19(6):402-8.

82. Jablonski-Jaudon RA, Kolanowski AM, Winstead V, Jones-Townsend C, Azuero A. Maturation of the MOUTh Intervention. Journal of Gerontological Nursing. 2016;42(3):15-23.

83. Janes NS, S.; Cott, C.; Rappolt, S. Figuring it out in the moment: A theory of unregulated care providers' knowledge utilization in dementia care settings. Worldviews on Evidence-Based Nursing. 2008;5(1):13-24.

84. Jenkins N, Keyes S, Strange L. Creating vignettes of early onset dementia: An exercise in public sociology. Sociology. 2016;50(1):77-92.

85. Jennings LA, Palimaru A, Corona MG, Cagigas XE, Ramirez KD, Zhao T, et al. Patient and caregiver goals for dementia care. Quality of Life Research: An International Journal of Quality of Life Aspects of Treatment, Care & Rehabilitation. 2017;26(3):685-93.

86. Johnston B, Lawton S, McCaw C, Law E, Murray J, Gibb J, et al. Living well with dementia: enhancing dignity and quality of life, using a novel intervention, Dignity Therapy. International Journal of Older People Nursing. 2016;11(2):107-20.

87. Kaufmann EG, Engel SA. Dementia and well-being: A conceptual framework based on Tom Kitwood’s model of needs. Dementia. 2016;15(4):774-88.

88. Kirkley CB, C.; Poole, M.; Arksey, H.; Hughes, J.; Bond, J. The impact of organisational culture on the delivery of person-centred care in services providing respite care and short breaks for people with dementia. Health and Social Care in the Community. 2011;19(4):438-48.

89. Kolanowski A, Van Haitsma K, Penrod J, Hill N, Yevchak A. Wish we would have known that!" Communication breakdown impedes person-centered care. Gerontologist. 2015;55:S50-S60.

90. Kreps D. Skeuomorphic Reassurance: Personhood and Dementia. In: Kreps D, Fletcher G, Griffiths M, editors. Technology and intimacy: choice or coercion. IFIP advances in information and communication technology. Cham: Springer; 2016. p. 61–71.

91. Lann-Wolcott HM, L. J.; Williams, K. Measuring the Person-Centeredness of Caregivers Working With Nursing Home Residents With Dementia. Behavior Therapy. 2011;42(1):89-99.

92. Leverton M, Burton A, Beresford-Dent J, Rapaport P, Manthorpe J, Azocar I, et al. Supporting independence at home for people living with dementia: a qualitative ethnographic study of homecare. Soc Psychiatry Psychiatr Epidemiol. 2021;56(12):2323-36.

93. Li J, Porock D. Resident outcomes of person-centered care in long-term care: A narrative review of interventional research. International Journal of Nursing Studies. 2014;51(10):1395–415.

94. Lopez RPM, K. M.; Mitchell, S. L.; Givens, J. L. What is family-centered care for nursing home residents with advanced dementia? American Journal of Alzheimer's Disease and other Dementias. 2013;28(8):763-8.

95. Manji S. Aging with dementia and an intellectual disability: A case study of supported empowerment in a community living home. US: ProQuest Information & Learning; 2009.

96. Manthorpe J, Samsi K. Person-centered dementia care: Current perspectives. Clinical Interventions in Aging. 2016;11:1733-40.

97. Martin G. Recovery approach to the care of people with dementia: Decision making and 'best interests' concerns. Journal of Psychiatric and Mental Health Nursing. 2009;16(7):654-60.

98. Martinez T, Suarez-Alvarez J, Yanguas J. Instruments for assessing Person Centered Care in Gerontology. Psicothema. 2016;28(2):114-21.

99. McAllister CLS, M. A. Community formation and community roles among persons with Alzheimer's disease: A comparative study of experiences in a residential Alzheimer's facility and a traditional nursing home. Qualitative Health Research. 1999;9(1):65-85.

100. McClendon MJS, K. A. Quality of informal care for persons with dementia: Dimensions and correlates. Aging and Mental Health. 2013;17(8):1003-15.

101. McIntyre M. Dignity in dementia: Person-centered care in community. Journal of Aging Studies. 2003;17(4):473-84.

102. Mitchell GT, Michelle. Ethical considerations of doll therapy for people with dementia. Nursing Ethics. 2014;21(6):720-30.

103. Morhardt DS, Marcia. From Person-Centered Care to Relational Centered Care. Generations. 2013;37(3):37-44.

104. Niemeijer ARD, M. F.; Frederiks, B. J.; Hertogh, C. M. The experiences of people with dementia and intellectual disabilities with surveillance technologies in residential care. Nursing ethics. 2014.

105. Nolan MRD, S.; Brown, J.; Keady, J.; Nolan, J. Beyond 'person-centred' care: a new vision for gerontological nursing. Journal of Clinical Nursing. 2004;13(3A):45-53.

106. Nowell ZCT, Amanda; Simpson, Jane. The subjective experience of personhood in dementia care settings. Dementia: The International Journal of Social Research and Practice. 2013;12(4):394-409.

107. O'Dwyer C. Official conceptualizations of person-centered care: Which person counts? Journal of Aging Studies. 2013;27(3):233-42.

108. O'Sullivan G. Ethical and effective: Approaches to residential care for people with dementia. Dementia. 2013;12(1):111-21.

109. Palmer JL. Preserving personhood of individuals with advanced dementia: Lessons from family caregivers. Geriatric Nursing. 2013;34(3):224-9.

110. Parker J. Interrogating person-centred dementia care in social work and social care practice. Journal of Social Work. 2001;1(3):329-45.

111. Powers BA. Everyday ethics of dementia care in nursing homes: A definition and taxonomy. American Journal of Alzheimer's Disease. 2000;15(3):143-51.

112. Reed PT, J. Dementia care practice recommendations for nursing homes and assisted living, phase 2: Falls, wandering, and physical restraints. Alzheimer's Care Today. 2008;9(1):51-9.

113. Robinson GEG, A. Culture change impacts quality of life for nursing home residents. Topics in Clinical Nutrition. 2008;23(2):120-30.

114. Robinson LB, C.; Briel, R.; Spencer, J.; Whitty, P. Improving patient-centered care for people with dementia in medical encounters: An educational intervention for old age psychiatrists. International Psychogeriatrics. 2010;22(1):129-38.

115. Robinson LH, D.; Corner, L.; Finch, T.; Hughes, J.; Brittain, K.; Bond, J. Balancing rights and risks: Conflicting perspectives in the management of wandering in dementia. Health, Risk and Society. 2007;9(4):389-406.

116. Rokstad AMME, K.; Edvardsson, D.; Selbæk, G. Psychometric evaluation of the Norwegian version of the Person-centred Care Assessment Tool. International Journal of Nursing Practice. 2012;18(1):99-105.

117. Rondon-Sulbaran J, Daly Lynn J, McCormack B, Ryan A, Martin S. The transition to technology-enriched supported accommodation (TESA) for people living with dementia: the experience of formal carers. Ageing & Society. 2020;40(10):2287-308.

118. Round JS, E. L.; Jones, L. A framework for understanding quality of life in individuals without capacity. Quality of Life Research. 2014;23(2):477-84.

119. Rushton C, Edvardsson D. Reconciling concepts of space and person-centred care of the older person with cognitive impairment in the acute care setting. Nursing Philosophy. 2017;18(3).

120. Sauer PEF-L, J.; Kinney, J. M.; Lokon, E. "It makes me feel like myself": Person-centered versus traditional visual arts activities for people with dementia. Dementia (London, England). 2014.

121. Savundranayagam MY, Moore-Nielsen K. Language-based communication strategies that support person-centered communication with persons with dementia. International Psychogeriatrics. 2015;27(10):1707-18.

122. Schwartz DBD, A.; Goldman, B.; Gramigna, G. D.; Cummings, B. Achieving Patient-Centered Care in a Case of a Patient With Advanced Dementia. Nutrition in Clinical Practice. 2014;29(4):556-8.

123. Sinclair C, Bajic-Smith J, Gresham M, Blake M, Bucks RS, Field S, et al. Professionals’ views and experiences in supporting decision-making involvement for people living with dementia. Dementia. 2021;20(1):84-105.

124. Smebye KLK, M. The influence of relationships on personhood in dementia care: A qualitative, hermeneutic study. BMC Nursing. 2013;12(1).

125. Snoeren MMWCJ, B. M.; Niessen, T. J. H.; Abma, T. A. Nurturing Cultural Change in Care for Older People: Seeing the Cherry Tree Blossom. Health Care Analysis. 2014.

126. Snowdon J. Psychogeriatric services in the community and in long-term care facilities: Needs and developments. Current Opinion in Psychiatry. 2007;20(6):533-8.

127. Stechl EL, G.; Steinhagen-Thiessen, E.; Flick, U. Subjektive Wahrnehmung und Bewältigung der Demenz im Frühstadium - SUWADEM. Eine qualitative Interviewstudie mit Betroffenen und Angehörigen. Zeitschrift für Gerontologie und Geriatrie. 2007;40(2):71-80.

128. Sugihara TF, T.; Phaal, R.; Ikawa, Y. A technology roadmap of assistive technologies for dementia care in Japan. Dementia (London, England). 2013.

129. Swinnen AMC. Healing words: A study of poetry interventions in dementia care. Dementia. 2016;15(6):1377-404.

130. Teng C, Sellars M, Pond D, Latt MD, Waite LM, Sinka V, et al. Making decisions about long-term institutional care placement among people with dementia and their caregivers: Systematic review of qualitative studies. Gerontologist. 2020;60(4):e329-e46.

131. Tetrault A, Nyback MH, Vaartio-Rajalin H, Fagerström L. Advance care planning in dementia care: Wants, beliefs, and insight. Nurs Ethics. 2022;29(3):696-708.

132. Thomsen MH, Michael; Müller-Hergl, Christian. Vertrauensvolle Begegnungen fördern. Pflegezeitschrift. 2010;Jg. 63, Nr. 11, S. 672-675 : Abb., Lit.:237.

133. Thornton L. Person-centred dementia care: an essential component of ethical nursing care. Canadian Nursing Home. 2011;22(3):10-4.

134. Vernooij-Dassen M, Moniz-Cook E, Jeon Y-H. Social health in dementia care: harnessing an applied research agenda. International Psychogeriatrics. 2018;30(6):775-8.

135. Viau-Guay AB, M.; Feillou, I.; Trudel, L.; Desrosiers, J.; Robitaille, M. J. Person-centered care training in long-term care settings: Usefulness and facility of transfer into practice. Canadian Journal on Aging. 2013;32(1):57-72.

136. Watson J. Developing the Senses Framework to support relationship-centred care for people with advanced dementia until the end of life in care homes. Dementia (14713012). 2019;18(2):545-66.

137. White DLN-C, L.; Lyons, K. S. Development and initial testing of a measure of person-directed care. Gerontologist. 2008;48:114-23.

138. Wilberforce M, Challis D, Davies L, Kelly MP, Roberts C, Clarkson P. Person-centredness in the community care of older people: A literature-based concept synthesis. International Journal of Social Welfare. 2017;26(1):86-98.

139. Williams J, Hadjistavropoulos T, Ghandehari OO, Yao X, Lix L. An evaluation of a person-centred care programme for long-term care facilities. Ageing and Society. 2015;35(3):457-88.

140. Williams KH, B.; Lueger, A.; Ward, K.; Wassmer, R.; Weber, A. Visual Cues for Person-centered Communication. Clinical Nursing Research. 2011;20(4):448-61.

141. Wolfe SE, Greenhill B, Butchard S, Day J. The meaning of autonomy when living with dementia: A Q-method investigation. Dementia. 2021;20(6):1875-90.
