# Supplementary material for: Autonomy and its relevance for the construction of personhood in dementia– a thematic synthesis
Source: BMC Geriatr. 2024 Mar 14;24:255. doi: 10.1186/s12877-024-04808-6 (PMC10941450; doi:10.1186/s12877-024-04808-6)
Supplement: Supplementary file 3 — Supplementary Material 3 [file 12877_2024_4808_MOESM3_ESM.docx]

**Appendix 3: Table S3**

In the following table, the analytical themes and the detailed description derived from the analysis are displayed.

| **Analytical Theme *Losing autonomy*** | | |
| --- | --- | --- |
| **Detailed Description** | | |
| One article generally states that people living with dementia are at increased risk of loss of autonomy and independence (1) or, more specific, at increased risk of losing autonomy by losing their familiar living environment (2). Others discuss that with the progression of the disease, the autonomy of people with dementia declines (3, 4), even to the point where it is nigh impossible for them to act autonomously in the severe stages. | Other authors state that lack of competence or limited mental capabilities threatens the autonomy of people living with dementia (5-7). Additionally, some mention a lack of decision-making ability, which leads, on the one hand, to the appointment of surrogate decision makers (8-10) and, on the other hand, in the scientific field simply to exclusion from research (11). | Moreover, some discuss that dementia reduces the capacity for self-determination (12, 13) or that the loss of autonomy is caused by cognitive degradation (14, 15). We found one example that claims that medication can be used to address the loss of autonomy or independence caused by neuropsychiatric symptoms (16). |
| The loss of autonomy has different consequences. On the one hand, we found examples in which the consequences of losing autonomy were discussed as depending on cultural context. We found an understanding that dementia leads to a loss of autonomy, but the consequences of this assumption are discussed as culturally contingent (17, 18). We also found the contradictory statement that the erosion of personhood does not depend on the cultural understanding of the person (3). | Furthermore, – and this argument is even more present – we found the discussion of the notion that losing autonomy directly threatens the personhood of people living with dementia (3, 6, 19). One perspective – referred to as a legal debate – describes a specific understanding of the legal person that sets typical characteristics and attributes for being a person that are hardly achievable for people with dementia (5). | Others discuss the notion that rational decision-making and being autonomous are typical aspects of personhood; functional and cognitive deficits exclude people with dementia from having full personhood in that sense (17, 20). Behuniak (5) argues against a conception of personhood that assumes cognitive capacity as a prerequisite and therefore excludes people with dementia from social participation. This argument is considered an effect of a culture of extreme individualism (14) and the establishment of a dominant reductive biomedical view (21). Doyle (22) presents an example of othering in which the personhood of people living with dementia is threatened by a perceived loss of autonomy. |

| **Analytical Theme *Assisting autonomy*** | | |
| --- | --- | --- |
| **Detailed Description** | | |
| In the literature, one can find the relatively nonspecific statement that assisting autonomy is possible (11, 22-24). More specifically, we found statements that autonomy can be promoted through others (15, 25-27) and the general idea that autonomy is associated with personhood (28, 29). Fazio (30), Mitchell and Templeton (9) and Boumans and colleagues (31) point out that there are different levels of autonomy and different levels of complexity of decisions in the case of dementia. | Moreover, some texts state that supporting autonomy is a central part of person-centred care (13, 21, 22, 26, 30-36). Autonomy is also described as one explicit aim of person-centred care (37-40). Within the scope of person-centred care models, we identified different connotations, for example, that autonomy is part of resident-centred care (38) and person-directed care (41, 42). Inter alia, Irving and Lakeman (43) state that autonomy can be maintained through the model of recovery-focused care. Aasgaard and colleagues (44) and Jablonski-Jaudon et al. (45) mention that autonomy is related to person-centred care. Rokstad et al. (46) and Martinez and colleagues (41) consider autonomy to be part of person-centred care measurement tools. Additionally, in the context of person-centred care, Dupuis et al. (47) mentions that autonomy is an aim of the culture change movement, and Robinson and Gallagher (48) state that autonomy is provided by the Green House Project. Occupational therapy is seen as one vehicle for emphasizing autonomy (49). McClendon and Smyth (50) see supporting autonomy as part of providing high-quality care. Individual personhood care is another approach that preserves autonomy (51). Robinson et al. (52) recognize autonomy as a core component of all types of centeredness. Autonomy is understood as part of individualized care by Martinez et al. (41). Ahessy (53) mentions that person-centred music therapy supports autonomy. In person-centred community-based care services, partnerships between practitioners and family carers are required to promote autonomy (49). According to Aasgaard and colleagues (44), person-centred care is realized through the promotion of autonomy. Wilberforce et al. (54) states that determination and self-planning supported by care through the concerned individuals themselves lead to autonomy. In contrast, we also found statements that the concept of autonomy is not part of person-centred care (55-57) or that autonomy is not a focus of relationship-centred care (58). | Another point is the explication of how to assist autonomy, as in Lann-Wolcott and colleagues (59) description of a general paradigm shift – under the heading “culture change” – in dementia care towards more flexibility and self-determination. Boumans et al. (26, 31), Estrada (34) and Hennelly et al. (60) refer to the relationship between people living with dementia and the formal and informal caregivers as one of the main element for maintaining autonomy and the way how the first are approached by the formal caregivers during care and welfare activities. More specifically, several authors mention knowledge of people living with dementia and biography-based assistance, which lead to more autonomy (11, 55, 61-63). Individual knowledge is also relevant when engaging people living with dementia in activities that they preferred prior to their diagnosis to maintain a sense of autonomy (49, 64, 65) and with regard to advanced directives (66, 67). Furthermore, we found statements that autonomy can be supported by allowing choice to people living with dementia or involving them in decisions (11, 19, 30, 36, 54, 66, 68, 69). Similarly, providing persons concerned with appropriate information to enable them to participate in decision-making processes is mentioned as a way to support their autonomy (52, 54, 70, 71). Listening to and communicating with people living with dementia is another way to support autonomy (63, 70, 72). Some texts note that autonomy can be sustained by allowing people living with dementia to be autonomous or act autonomously through their social environment (11, 30, 73, 74). Some authors find that focusing on the remaining resources is necessary to support autonomy for people living with dementia (6, 11, 21). Vernooij-Dassen et al. (75) points out that the assistance of people living with dementia to use their capacities can be realized through an assessment of person-environment fit, person-directed goal setting and interdisciplinary team support to maintain autonomy. Others identify allowing time to act on decisions as a crucial factor for promoting greater autonomy (19, 76, 77). Respecting the person’s wishes is also a way to promote autonomy (64, 78), as is formalizing such wishes in formal contracts with care providers (11). Martinez et al. (41) points out that with regard to difficulties in meeting the preferences of people living with dementia, professional carers are in charge and must use strategies of observation and representation to support the indirect exercise of autonomy. Chung and colleagues (64) see autonomy as part of sustaining agency by engaging the persons concerned in daily activities. The concept of patient participation is related to the patient’s autonomy and is mentioned by Helgesen et al. (79) as one way to assist autonomy. Cayton (80) sees a connection between a combination of earlier diagnosis, symptomatic drug treatment and being active and self-determinant. |
| Another topic identified in the included literature is the potential influence of institutional requirements on the autonomy of people living with dementia. Some authors describe institutional requirements that actually restrict the autonomy of people living with dementia (2, 11, 22, 63, 81, 82). Others state that the behaviour and actions of staff and the institutional organization restrict the autonomy of people living with dementia (60, 68, 83). Another influencing aspect has been described with regard to the environment, whereby institutional routines might restrict autonomy (11, 15, 19, 65, 82). Doyle (22), Hunter et al. (84) and Boumans et al. (26, 31) mention that the physical environment not restricts but only influences the autonomy of people living with dementia. Fazio et al. (35) state a supportive community as a positive influencing factor as well as Leverton et al. (85) find the same in the home environment. Bergland and colleagues (86) and O’Sullivan (87) find that more autonomy is possible in a safe physical environment, and McAllister and Silverman (88) state that it is possible to facilitate independence and autonomy through specially designed environments. | Niemeijer and colleagues (89) point out that staff intervention can overcome institutional restrictions. In terms of institutional practice, an autonomy-respecting ethos promotes the autonomy of residents (90). Reed and Tilly (24) point out that the practice of using restraints impedes autonomy, and Bergland and colleagues (86) state that the imbalance of a person’s competencies and requirements (pressure and demands) with the environment influences autonomy. | Ericson and colleagues (76) state that keeping people at home is a primary motivation for caring and a major constituent of best care because it promotes greater autonomy as well as an essential sense of security for the person. |
| Regarding professional care, several aspects with reference to autonomy were found in the literature. First, specific interventions that are likely to support autonomy are mentioned (3, 22, 91-93). Additionally, Viau-Guay et al. (93) mention practical approaches (e.g., mobilizing residents’ capacities and being responsive to residents’ feedback or vertical mobilization) to foster the autonomy of people living with dementia. Manji (11) and Powers (69) find that autonomy can be supported through reflections or through reflective practice in care. Boumans and colleagues (31) found two mechanisms (flexibility and social inclusion) that explain which attitudes of formal caregivers are of importance to foster the experience of autonomy. Another way to evoke autonomy is to allow people living with dementia and their relatives to formulate care plans on their own instead of professionals (3, 11). Viau-Guay et al. (93) state that consensus between the different caring professions is an important prerequisite for maintaining and developing autonomy. | In addition to the possibility of assistance, we found notions of barriers to assisting autonomy, which we differentiated into two aspects. First, autonomy might be in conflict with other goals. We found assumptions that autonomy might be in conflict with care or organizational requirements (68, 69, 93), for example, hygienic care (22, 93), or with the protection of the will of others (94, 95). A major aspect is the challenge of balancing possible risks and the need for autonomy (5, 6, 88, 96-98). This involves topics such as self-harm or harm from or directed to others (11, 69, 99, 100), mobility (101), assisted living environments (24, 65, 102), risks of falling (22), wandering and the use of tagging devices (99), driving (64, 96), home hazards and financial affairs (103). | Among other influencing factors, studies have found that physical independence (22, 65) and the support of physical health (11, 22) lead to more autonomy. Some authors see the use of (surveillance) technology as a way to support autonomy (26, 31, 36, 39, 89, 99, 102), and Robinson and colleagues (99) specifically speak of the use of technology to solve the problem of conflicting care goals such as risk management and wandering. Hughes (7) and Robinson et al. (99) express a critical perspective on technology in the context of autonomy, for example, the risk of invading privacy or deepening the isolation of users or that technology is used to facilitate care processes and not explicitly maximize the person’s autonomy. |

| **Analytical Theme *Autonomy and Stigma*** | | |
| --- | --- | --- |
| **Detailed Description** | | |
| First, we found specific ideas or imaginations on people living with dementia that arise in professional models. We found the notion that models grounded in medical principles do not recognize issues of person-centeredness and client autonomy (104) or that applied care models view people living with dementia as dependent patients, which undermines their citizenship (5). Statements regarding the perception of people living with dementia are made by Hennelly et al. (60) who state that much of dementia care is oppressive because it is not based on mutual values leading to a diminution of autonomy and Heggestad et al. (63), who points towards the possibility of the dementia diagnosis becoming the “master status” of a person. As a consequence of this “master status”, the autonomy and self-determination of people living with dementia is overlaid by assumptions about the disease rather than being based on interactional experiences. Palmer (105) notes the concession that to act autonomously depends on the established culture of care in a professional context. Positive examples with regard to stigmatization processes and self-perception appear in Hilgeman et al. (106), who conclude that it is also possible to interpret the behaviours of people living with dementia as strengths rather than losses. Watson (107) refers to the point that embodied expressions of autonomy should be taken seriously as communicative, interactive, informative and intentional. | Second, some statements focus not only on the perception but also on the effective actions that follow. On the one hand (focusing on perception and the following action), we found examples of how diagnosis leads to exclusion (5, 11). Another point is that people living with dementia are denied autonomy despite having remaining abilities (6, 22), that others assume inability even though they may have retained ability (108) or, more generally, that determining if someone have autonomous capacities often depends on professionals (27). Other authors see culture shaping interactions so that people living with dementia are regarded as incapable of decision-making (70) or find that a so-called hypercognitive culture leads to labelling processes that neglect the moral status and remaining capabilities of the people concerned (66). One point is the distinction between “perceived” and “lived” impairment to express processes of pathologizing and expectations of people living with dementia (81). On the other hand (focusing more on the actions that follow), Manji (11) and Teng et al. (109) explicate the connection between the relational behaviour of others and the loss of autonomy and personhood. Others describe how dignity of identity (which includes autonomy) is threatened by forms of relationality (15, 110). | In terms of the possible reactions of people living with dementia to stigmatization, Stechl et al. (100) and Teng et al. (109) find examples of the people concerned actively defending their autonomy against stigmatization and external influences. Wolfe et al. (19) found the statement of a person living with dementia who expresses difficulty with the idea that other people influencing or taking decisions for him and sees this as an inevitable but dreaded consequence. |

| **Analytical Theme *Relevance of autonomy*** | | |
| --- | --- | --- |
| **Detailed Description** | | |
| In the discourses on the shift of relevance of the concept of autonomy in the context of dementia, we found several statements that autonomy is still important (48, 65, 67, 103, 111) or even more important since there is a shift in focus away from the traditional biomedical model (35). In more detail, Førsund et al. (2) state autonomy as one important element of the experience of lived space and Johnston et al. (112) and Kaufmann and Engel (113) note that autonomy defines identity, and Dewing (83) states the same for the concept of personhood. Manji (11) and Sauer et al. (25) see autonomy as an important concept for the quality of life of people living with dementia, and Kaufmann and Engel (113) believe that mental health is important. Autonomy is important in reinforcing the self, as noted by Wilberforce and colleagues (54), and Dupuis et al. (47) include autonomy in the concept of citizenship. | Some authors see other values or aspects as more important than autonomy in the context of dementia (19, 114). More specifically, personhood, interdependence and choice are rated as more important (18, 54, 55). Additionally, values such as respect, feeling at home and comfort are rated as more important than autonomy and choice (115). Behuniak (5), Downs et al. (116) and Lopez et al. (117) focus on interconnectedness rather than emphasizing independence or autonomy. Additionally, Hughes and Beatty (118) and Nolan et al. (55) challenge the primacy of autonomy by emphasizing concepts such as mutual dependency and authentic partnership. Inter alia, with reference to Harré, Higgs and Gilleard (114) point out that concepts such as agency, autonomy and personal authority are important for only one aspect of selfhood, whereas other aspects of selfhood are represented by social identities or socially constructed selves. Furthermore, Higgs and Gilleard (114) discuss the Kantian notion that an individual is a rights holder and persons are ends in themselves and finds that it reflects how to replace the construction of personhood with terms such as rationality, judgement and autonomy. With reference to Kitwood, Higgs and Gilleard (114) criticise the reduction of the concept of personhood to the criteria of autonomy and rationality and pleads for a conceptualization grounded on the principles of relationships and moral solidarity. In another interpretation of Kitwood, Heggestad et al. (110) criticise the emphasis on autonomy, individuality and successful ageing as goals of person-centred care. This new form of ageism can be countered with the construct of relationship-centred care, which sees every person as involved in social relations rather than focusing on individual, independent human beings. | At the level of the individual, we found statements that discuss the contingency of the concept in expressing different needs for autonomy (17, 34, 119). Stechl et al. (100), Kaufmann and Engel (113) and Hennelly et al. (60) formulate autonomy as an individually varying need, and Hughes and colleagues (58) state that it is not contradictory to have needs for autonomy and social embeddedness at the same time. |
| In contrast to the aforementioned studies, we found a critical view on individualistic perspectives from the standpoint of cultural criticism that states that the concept of autonomy has different levels of social importance (17, 120). Three articles express a critical view on rationality and autonomy in Western societies (14, 81, 121). O’Dwyer (56) and Wilberforce et al. (54) criticise how autonomy and person-centeredness are promoted in Western societies and find that they hinder relations because they overemphasize an individualistic perspective. Others assert that Western developments such as the disability movement and consumerism call for choice and highlight autonomy (9, 56, 58). |  |  |

| **Analytical Theme *Relationship-based autonomy*** | | |
| --- | --- | --- |
| **Detailed Description** | | |
| Regarding the idea that autonomy emerges in social relations rather than being an individualistic conceptualization, we detected several discussions in the literature that autonomy takes place in social relations. Some studies state that it is a fallacy that people make and act on key decisions on their own or in isolation from their networks (70, 122). Another statement, oriented on the Nuffield Council on Bioethics, claims a different concept of the person and instead recommends a “situated embodied agent” model that emphasizes embeddedness and social dependency in a construct of relational autonomy (7). Similarly, Niemeijer et al. (89) criticise the focus on independent persons in questions of decision-making and urges the recognition of the complex conditions in long-term care. | Nolan et al. (55), with reference to MacDonald, Clark, McCormack and Mulrooney, support notions of relational autonomy with a focus on social relationships, interconnectedness and interdependence when speaking of high-quality care. Moreover, he criticizes the notion that therapeutic nursing care cannot be based on an individualistic concept of autonomy because it is based on the individual relationship between nurses and patients (55). | Heggestad et al. (110) refer to the debate on human dignity by recommending a relational view on autonomy that focuses on interdependence and social circumstances and simultaneously confirms that people living with dementia are equal human beings. Finally, Manthorpe and Samsi (123), Tetrault et al. (13) and Wolfe et al. (19) refocus on relationships in care and relationship-based autonomy. |

**References**

1. Cruz J, Marques A, Barbosa A, Figueiredo D, Sousa LX. Making sense(s) in dementia: A multisensory and motor-based group activity program. American Journal of Alzheimer's Disease and Other Dementias. 2013;28(2):137-46.

2. Førsund LH, Grov EK, Helvik AS, Juvet LK, Skovdahl K, Eriksen S. The experience of lived space in persons with dementia: A systematic meta-synthesis. BMC Geriatr. 2018;18(1).

3. De Waal H. Rethinking dementia: How autonomy and control can be fostered through the development of person centred services. Working with Older People. 2014;18(2):82-9.

4. Round J, Sampson EL, Jones L. A framework for understanding quality of life in individuals without capacity. Quality of Life Research. 2014;23(2):477-84.

5. Behuniak SM. Toward a political model of dementia: Power as compassionate care. Journal of Aging Studies. 2010;24(4):231-40.

6. Dewing J. Dementia. Part 1: Person-centred care. Professional nurse (London, England). 1999;14(8):585-8; quiz 3-4.

7. Hughes JC. Philosophical issues in dementia. Current Opinion in Psychiatry. 2013;26(3):283-8.

8. Martin G. Recovery approach to the care of people with dementia: Decision making and 'best interests' concerns. Journal of Psychiatric and Mental Health Nursing. 2009;16(7):654-60.

9. Mitchell G, Templeton M. Ethical considerations of doll therapy for people with dementia. Nursing Ethics. 2014;21(6):720-30.

10. Schwartz DB, DiTucci A, Goldman B, Gramigna GD, Cummings B. Achieving Patient-Centered Care in a Case of a Patient With Advanced Dementia. Nutrition in Clinical Practice. 2014;29(4):556-8.

11. Manji S. Aging with dementia and an intellectual disability: A case study of supported empowerment in a community living home. US: ProQuest Information & Learning; 2009.

12. Chenoweth L, King M, Luscombe G, Forbes I, Jeon YH, Parbury JS, et al. Study Protocol of a Randomised Controlled Group Trial of Client and Care Outcomes in the Residential Dementia Care Setting. Worldviews on Evidence-Based Nursing. 2011;8(3):153-65.

13. Tetrault A, Nyback MH, Vaartio-Rajalin H, Fagerström L. Advance care planning in dementia care: Wants, beliefs, and insight. Nurs Ethics. 2022;29(3):696-708.

14. Ames S. What happens to the person with dementia? Journal of Religion, Spirituality & Aging. 2016;28(1-2):118–35.

15. Bentwich ME, Dickman N, Oberman A. Human dignity and autonomy in the care for patients with dementia: Differences among formal caretakers from various cultural backgrounds. Ethnicity & health. 2016:1–21.

16. Hoe J, Thompson R. Promoting positive approaches to dementia care in nursing. Nursing Standard. 2010;25(4):47-56.

17. Castillo EH. Doing dementia better: Anthropological insights. Clinics in Geriatric Medicine. 2011;27(2):273-89.

18. Thomsen M, Herrmann M, Müller-Hergl C. Vertrauensvolle Begegnungen fördern. Pflegezeitschrift. 2010;Jg. 63, Nr. 11, S. 672-675 : Abb., Lit.:237.

19. Wolfe SE, Greenhill B, Butchard S, Day J. The meaning of autonomy when living with dementia: A Q-method investigation. Dementia. 2021;20(6):1875-90.

20. Parker J. Interrogating person-centred dementia care in social work and social care practice. Journal of Social Work. 2001;1(3):329-45.

21. Morhardt D, Spira M. From Person-Centered Care to Relational Centered Care. Generations. 2013;37(3):37-44.

22. Doyle PJ. Definitions, interpretations, and uses of person-centered care in a dementia-specific long-term care setting: A cultural analysis. US: ProQuest Information & Learning; 2012.

23. Nowell ZC, Thornton A, Simpson J. The subjective experience of personhood in dementia care settings. Dementia: The International Journal of Social Research and Practice. 2013;12(4):394-409.

24. Reed P, Tilly J. Dementia care practice recommendations for nursing homes and assisted living, phase 2: Falls, wandering, and physical restraints. Alzheimer's Care Today. 2008;9(1):51-9.

25. Sauer PE, Fopma-Loy J, Kinney JM, Lokon E. "It makes me feel like myself": Person-centered versus traditional visual arts activities for people with dementia. Dementia (London). 2014.

26. Boumans J, van Boekel LC, Verbiest MEA, Baan CA, Luijkx KG. Exploring how residential care facilities can enhance the autonomy of people with dementia and improve informal care. Dementia. 2022;21(1):136-52.

27. Sinclair C, Bajic-Smith J, Gresham M, Blake M, Bucks RS, Field S, et al. Professionals’ views and experiences in supporting decision-making involvement for people living with dementia. Dementia. 2021;20(1):84-105.

28. Buron B. Levels of Personhood: A Model for Dementia Care. Geriatric Nursing. 2008;29(5):324-32.

29. Edvardsson D, Fetherstonhaugh D, Nay R. Promoting a continuation of self and normality: person-centred care as described by people with dementia, their family members and aged care staff. Journal of Clinical Nursing. 2010;19(17/18):2611-8.

30. Fazio S. The enduring self in people with Alzheimer's: Getting to the heart of individualized care. Baltimore, MD, US: Health Professions Press; 2008.

31. Boumans J, Van Boekel LC, Baan CA, Luijkx KG, Heyn PC. How Can Autonomy Be Maintained and Informal Care Improved for People with Dementia Living in Residential Care Facilities: A Systematic Literature Review. Gerontologist. 2019;59(6):E709-E30.

32. Colomer J, de Vries J. Person-centred dementia care: a reality check in two nursing homes in Ireland. Dementia. 2016;15(5):1158-70.

33. Downs M. Putting People-and Compassion-First: The United Kingdom's Approach to Person-Centered Care for Individuals with Dementia. Generations. 2013;37(3):53-9.

34. Estrada R. Listen and You Will See the Person Through the Dementia. J Pers Oriented Res. 2021;7(2):88-97.

35. Fazio S, Pace D, Flinner J, Kallmyer B. The Fundamentals of Person-Centered Care for Individuals with Dementia. Gerontologist. 2018;58:S10-S9.

36. Rondon-Sulbaran J, Daly Lynn J, McCormack B, Ryan A, Martin S. The transition to technology-enriched supported accommodation (TESA) for people living with dementia: the experience of formal carers. Ageing & Society. 2020;40(10):2287-308.

37. Brown Wilson C, Swarbrick C, Pilling M, Keady J. The senses in practice: Enhancing the quality of care for residents with dementia in care homes. Journal of Advanced Nursing. 2013;69(1):77-90.

38. Li J, Porock D. Resident outcomes of person-centered care in long-term care: A narrative review of interventional research. International Journal of Nursing Studies. 2014;51(10):1395–415.

39. Sugihara T, Fujinami T, Phaal R, Ikawa Y. A technology roadmap of assistive technologies for dementia care in Japan. Dementia (London). 2013.

40. Williams K, Harris B, Lueger A, Ward K, Wassmer R, Weber A. Visual Cues for Person-centered Communication. Clinical Nursing Research. 2011;20(4):448-61.

41. Martinez T, Suarez-Alvarez J, Yanguas J. Instruments for assessing Person Centered Care in Gerontology. Psicothema. 2016;28(2):114-21.

42. White DL, Newton-Curtis L, Lyons KS. Development and initial testing of a measure of person-directed care. Gerontologist. 2008;48:114-23.

43. Irving K, Lakeman R. Reconciling mental health recovery with screening and early intervention in dementia care. International Journal of Mental Health Nursing. 2010;19(6):402-8.

44. Aasgaard HS, Fagerstrom L, Landmark B. Nurses’ Experiences of Providing Care to Dementia Patients Through Home Health Care: After Further Training and a Reorganization of Nursing Resources. Home Health Care Management & Practice. 2014;26(4):230-8.

45. Jablonski-Jaudon RA, Kolanowski AM, Winstead V, Jones-Townsend C, Azuero A. Maturation of the MOUTh Intervention. Journal of Gerontological Nursing. 2016;42(3):15-23.

46. Rokstad AMM, Engedal K, Edvardsson D, Selbæk G. Psychometric evaluation of the Norwegian version of the Person-centred Care Assessment Tool. International Journal of Nursing Practice. 2012;18(1):99-105.

47. Dupuis S, McAiney CA, Fortune D, Ploeg J, Witt LD. Theoretical foundations guiding culture change: The work of the Partnerships in Dementia Care Alliance. Dementia. 2016;15(1):85-105.

48. Robinson GE, Gallagher A. Culture change impacts quality of life for nursing home residents. Topics in Clinical Nutrition. 2008;23(2):120-30.

49. Chung P. Professionals partnering with family carers in home-based activity for those with dementia. WFOT Bulletin. 2013;67:9-16.

50. McClendon MJ, Smyth KA. Quality of informal care for persons with dementia: Dimensions and correlates. Aging and Mental Health. 2013;17(8):1003-15.

51. Thornton L. Person-centred dementia care: an essential component of ethical nursing care. Canadian Nursing Home. 2011;22(3):10-4.

52. Robinson L, Bamford C, Briel R, Spencer J, Whitty P. Improving patient-centered care for people with dementia in medical encounters: An educational intervention for old age psychiatrists. International Psychogeriatrics. 2010;22(1):129-38.

53. Ahessy B. Song writing with clients who have dementia: A case study. Arts in Psychotherapy. 2017;55:23–31.

54. Wilberforce M, Challis D, Davies L, Kelly MP, Roberts C, Clarkson P. Person-centredness in the community care of older people: A literature-based concept synthesis. International Journal of Social Welfare. 2017;26(1):86-98.

55. Nolan MR, Davies S, Brown J, Keady J, Nolan J. Beyond 'person-centred' care: a new vision for gerontological nursing. Journal of Clinical Nursing. 2004;13(3A):45-53.

56. O'Dwyer C. Official conceptualizations of person-centered care: Which person counts? Journal of Aging Studies. 2013;27(3):233-42.

57. Swinnen AMC. Healing words: A study of poetry interventions in dementia care. Dementia. 2016;15(6):1377-404.

58. Hughes JC, Bamford C, May C. Types of centredness in health care: themes and concepts. Medicine Health Care and Philosophy. 2008;11(4):455-63.

59. Lann-Wolcott H, Medvene LJ, Williams K. Measuring the Person-Centeredness of Caregivers Working With Nursing Home Residents With Dementia. Behavior Therapy. 2011;42(1):89-99.

60. Hennelly N, Cooney A, Houghton C, O'Shea E. Personhood and Dementia Care: A Qualitative Evidence Synthesis of the Perspectives of People with Dementia. Gerontologist. 2021;61(3):E85-E100.

61. Edvardsson D, Innes A. Measuring person-centered care: A critical comparative review of published tools. Gerontologist. 2010;50(6):834-46.

62. Gilpin L. The Planetree Model: Its impact on caring for those with dementia. Alzheimer's Care Quarterly. 2006;7(4):273-7.

63. Heggestad AKT, Nortvedt P, Slettebo A. 'Like a prison without bars': Dementia and experiences of dignity. Nursing Ethics. 2013;20(8):881-92.

64. Chung P, Ellis-Hill C, Coleman P. Supporting activity engagement by family carers at home: maintenance of agency and personhood in dementia. Int J Qual Stud Health Well-being. 2017;12(1):1267316.

65. Frankowski AC, Clark LJ. Sexuality and Intimacy in Assisted Living: Residents' Perspectives and Experiences. Sexuality Research and Social Policy. 2009;6(4):25-37.

66. Dewing J. From ritual to relationship: A person-centred approach to consent in qualitative research with older people who have dementia. Dementia: The International Journal of Social Research and Practice. 2002;1(2):157-71.

67. Hughes JC. Conceptual issues in 'cognitive impairment'. Current Opinion in Psychiatry. 2015;28(2):188–93.

68. Cooney A, Hunter A, Murphy K, Casey D, Devane D, Smyth S, et al. 'Seeing me through my memories': A grounded theory study on using reminiscence with people with dementia living in long-term care. Journal of Clinical Nursing. 2014.

69. Powers BA. Everyday ethics of dementia care in nursing homes: A definition and taxonomy. American Journal of Alzheimer's Disease. 2000;15(3):143-51.

70. Clarke CL, Alexjuk J, Gibb CE. Information in dementia care: Sense making and a public health direction for the UK? International Journal of Older People Nursing. 2011;6(3):237-43.

71. Hajime T, Atsuko K, Tomoko K, Hiroko Y. Relative Preservation of Advanced Activities in Daily Living among Patients withMild-to-Moderate Dementia in the Community and Overview of Support Provided by Family Caregivers. International Journal of Alzheimer's Disease. 2012:1-7.

72. Dewing J. Concerns relating to the application of frameworks to promote person-centredness in nursing with older people. Journal of Clinical Nursing. 2004;13(3 A):39-44.

73. Beerens HC, Zwakhalen SMG, Verbeek H, F EST, Jolani S, Downs M, et al. The relation between mood, activity, and interaction in long-term dementia care. Aging Ment Health. 2016:1-7.

74. Edvardsson D, Winblad B, Sandman P. Person-centred care of people with severe Alzheimer's disease: current status and ways forward. The Lancet Neurology. 2008;7(4):362-7.

75. Vernooij-Dassen M, Moniz-Cook E, Jeon Y-H. Social health in dementia care: harnessing an applied research agenda. International Psychogeriatrics. 2018;30(6):775-8.

76. Ericson I, Hellström I, Lundh U, Nolan M. What constitutes good care for people with dementia? British journal of nursing (Mark Allen Publishing). 2001;10(11):710-4.

77. Gridley K, Brooks J, Glendinning C. Good practice in social care for disabled adults and older people with severe and complex needs: Evidence from a scoping review. Health & Social Care in the Community. 2014;22(3):234-48.

78. Savundranayagam MY, Moore-Nielsen K. Language-based communication strategies that support person-centered communication with persons with dementia. International Psychogeriatrics. 2015;27(10):1707-18.

79. Helgesen AK, Larsson M, Athlin E. 'Patient participation' in everyday activities in special care units for persons with dementia in Norwegian nursing homes. International Journal of Older People Nursing. 2010;5(2):169-78.

80. Cayton H. Telling stories: choices and challenges on the journey of dementia. Dementia (14713012). 2004;3(1):9-17.

81. Aubrecht K, Keefe J. “Everybody has different levels of why they are here”: Deconstructing domestication in the nursing home setting. In: Rembis M, editor. Disabling Domesticity. New York: Palgrave Macmillan US; 2016. p. 215–39.

82. Clark-McGhee K, Castro M. A narrative analysis of poetry written from the words of people given a diagnosis of dementia. Dementia. 2015;14(1):9-26.

83. Dewing J. Personhood and dementia: Revisiting Tom Kitwood's ideas. International Journal of Older People Nursing. 2008;3(1):3-13.

84. Hunter PV, Hadjistavropoulos T, Thorpe L, Lix LM, Malloy DC. The influence of individual and organizational factors on person-centred dementia care. Aging & Mental Health. 2016;20(7):700-8.

85. Leverton M, Burton A, Beresford-Dent J, Rapaport P, Manthorpe J, Azocar I, et al. Supporting independence at home for people living with dementia: a qualitative ethnographic study of homecare. Soc Psychiatry Psychiatr Epidemiol. 2021;56(12):2323-36.

86. Bergland A, Kirkevold M, Edvardsson D. Psychometric properties of the Norwegian Person-centred Climate Questionnaire from a nursing home context. Scandinavian Journal of Caring Sciences. 2012;26(4):820-8.

87. O'Sullivan G. Ethical and effective: Approaches to residential care for people with dementia. Dementia. 2013;12(1):111-21.

88. McAllister CL, Silverman MA. Community formation and community roles among persons with Alzheimer's disease: A comparative study of experiences in a residential Alzheimer's facility and a traditional nursing home. Qualitative Health Research. 1999;9(1):65-85.

89. Niemeijer AR, Depla MF, Frederiks BJ, Hertogh CM. The experiences of people with dementia and intellectual disabilities with surveillance technologies in residential care. Nurs Ethics. 2014.

90. Gladman JRF, Jones RG, Radford K, Walker E, Rothera I. Person-centred dementia services are feasible, but can they be sustained? Age and Ageing. 2007;36(2):171-6.

91. Barbosa A, Nolan M, Sousa L, Marques A, Figueiredo D. Effects of a Psychoeducational Intervention for Direct Care Workers Caring for People With Dementia: results From a 6-Month Follow-Up Study. American journal of alzheimer's disease and other dementias [Internet]. 2016; 31(2):[144-55 pp.]. Available from: <http://onlinelibrary.wiley.com/o/cochrane/clcentral/articles/870/CN-01200870/frame.html>

<http://journals.sagepub.com/doi/pdf/10.1177/1533317515603500>.

92. Bryden C. A person-centred approach to counselling, psychotherapy and rehabilitation of people diagnosed with dementia in the early stages. Dementia (14713012). 2002;1(2):141-56.

93. Viau-Guay A, Bellemare M, Feillou I, Trudel L, Desrosiers J, Robitaille MJ. Person-centered care training in long-term care settings: Usefulness and facility of transfer into practice. Canadian Journal on Aging. 2013;32(1):57-72.

94. Fox M, Wilson L. Person-centered advocacy for people with dementia... first of two articles. Journal of Dementia Care. 2007;15(2):17-9.

95. Hirsch RD. Sozio- und Psychotherapie bei Alzheimerkranken. Zeitschrift für Gerontologie und Geriatrie. 2001;34(2):92-100.

96. de Witt L, Ploeg J. Caring for older people living alone with dementia: Healthcare professionals’ experiences. Dementia. 2016;15(2):221-38.

97. Jenkins N, Keyes S, Strange L. Creating vignettes of early onset dementia: An exercise in public sociology. Sociology. 2016;50(1):77-92.

98. Smebye KL, Kirkevold M. The influence of relationships on personhood in dementia care: A qualitative, hermeneutic study. BMC Nursing. 2013;12(1).

99. Robinson L, Hutchings D, Corner L, Finch T, Hughes J, Brittain K, et al. Balancing rights and risks: Conflicting perspectives in the management of wandering in dementia. Health, Risk and Society. 2007;9(4):389-406.

100. Stechl E, Lämmler G, Steinhagen-Thiessen E, Flick U. Subjektive Wahrnehmung und Bewältigung der Demenz im Frühstadium - SUWADEM. Eine qualitative Interviewstudie mit Betroffenen und Angehörigen. Zeitschrift für Gerontologie und Geriatrie. 2007;40(2):71-80.

101. Evans S, Fear T, Means R, Vallelly S. Supporting independence for people with dementia in extra care housing. Dementia: The International Journal of Social Research and Practice. 2007;6(1):144-5.

102. Godwin B. The ethical evaluation of assistive technology for practitioners: A checklist arising from a participatory study with people with dementia, family and professionals. Journal of Assistive Technologies. 2012;6(2):123-35.

103. Jennings LA, Palimaru A, Corona MG, Cagigas XE, Ramirez KD, Zhao T, et al. Patient and caregiver goals for dementia care. Quality of Life Research: An International Journal of Quality of Life Aspects of Treatment, Care & Rehabilitation. 2017;26(3):685-93.

104. Clarke J. Adverse factors and the mental health of older people: Implications for social policy and professional practice. Journal of Psychiatric and Mental Health Nursing. 2005;12(3):290-6.

105. Palmer JL. Preserving personhood of individuals with advanced dementia: Lessons from family caregivers. Geriatric Nursing. 2013;34(3):224-9.

106. Hilgeman MM, Allen RS, Snow AL, Durkin DW, DeCoster J, Burgio L. Preserving Identity and Planning for Advance Care (PIPAC): preliminary outcomes from a patient-centered intervention for individuals with mild dementia. Aging & Mental Health. 2014;18(4):411-24.

107. Watson J. Developing the Senses Framework to support relationship-centred care for people with advanced dementia until the end of life in care homes. Dementia (14713012). 2019;18(2):545-66.

108. Han A, Radel J. The Benefits of a Person-Centered Social Program for Community-Dwelling People with Dementia: Interpretative Phenomenological Analysis. Activities, Adaptation & Aging. 2017;41(1):47–71.

109. Teng C, Sellars M, Pond D, Latt MD, Waite LM, Sinka V, et al. Making decisions about long-term institutional care placement among people with dementia and their caregivers: Systematic review of qualitative studies. Gerontologist. 2020;60(4):e329-e46.

110. Heggestad AKT, Nortvedt P, Slettebø Å. Dignity and care for people with dementia living in nursing homes. Dementia. 2015;14(6):825-41.

111. Williams J, Hadjistavropoulos T, Ghandehari OO, Yao X, Lix L. An evaluation of a person-centred care programme for long-term care facilities. Ageing and Society. 2015;35(3):457-88.

112. Johnston B, Lawton S, McCaw C, Law E, Murray J, Gibb J, et al. Living well with dementia: enhancing dignity and quality of life, using a novel intervention, Dignity Therapy. International Journal of Older People Nursing. 2016;11(2):107-20.

113. Kaufmann EG, Engel SA. Dementia and well-being: A conceptual framework based on Tom Kitwood’s model of needs. Dementia. 2016;15(4):774-88.

114. Higgs P, Gilleard C. Interrogating personhood and dementia. Aging & Mental Health. 2016;20(8):773-80.

115. Chaudhury H, Hung L, Badger M. The role of physical environment in supporting person-centered dining in long-term care: A review of the literature. American Journal of Alzheimer's Disease and other Dementias. 2013;28(5):491-500.

116. Downs M, Small N, Froggatt K. Person-centred Care for People with Severe Dementia. In: Burns AW, Bengt, editor. Severe dementia. New York, NY, US: John Wiley & Sons Ltd; 2006. p. 193-204.

117. Lopez RP, Mazor KM, Mitchell SL, Givens JL. What is family-centered care for nursing home residents with advanced dementia? American Journal of Alzheimer's Disease and other Dementias. 2013;28(8):763-8.

118. Hughes JC, Beatty A. Understanding the person with dementia: A clinicophilosophical case discussion. Advances in Psychiatric Treatment. 2013;19(5):337-43.

119. Hilton C, Moniz-Cook E. Examining the personality dimensions of sociotropy and autonomy in older people with dementia: Their relevance to person centred care. Behavioural and Cognitive Psychotherapy. 2004;32(4):457-65.

120. McIntyre M. Dignity in dementia: Person-centered care in community. Journal of Aging Studies. 2003;17(4):473-84.

121. Chapman M, Philip J, Komesaroff P. A person-centred problem. Hum Soc Sci Comm. 2022;9(1).

122. Gilmour JA, Brannelly T. Representations of people with dementia - subaltern, person, citizen. Nursing Inquiry. 2010;17(3):240-7.

123. Manthorpe J, Samsi K. Person-centered dementia care: Current perspectives. Clinical Interventions in Aging. 2016;11:1733-40.
